# ${{\varvec{L}}}^{{\varvec{p}}}$-Solutions and Comparison Results for Lévy-Driven Backward Stochastic Differential Equations in a Monotonic, General Growth Setting

**DOI:** 10.1007/s10959-020-01056-3

**Published:** 2020-11-24

**Authors:** Stefan Kremsner, Alexander Steinicke

**Affiliations:** 1 Department of Mathematics, University of Graz, Graz, Austria; 2 Department of Mathematics, Montanuniversitaet Leoben, Leoben, Austria

**Keywords:** Backward stochastic differential equation, Lévy process, $L^p$-solutions, Predictable version, 60H10

## Abstract

We present a unified approach to $L^p$-solutions ($p > 1$) of multidimensional backward stochastic differential equations (BSDEs) driven by Lévy processes and more general filtrations. New existence, uniqueness and comparison results are obtained. The generator functions obey a time-dependent extended monotonicity (Osgood) condition in the *y*-variable and have general growth in *y*. Within this setting, the results generalize those of Royer, Yin and Mao, Yao, Kruse and Popier, and Geiss and Steinicke.

## Introduction

The existence and uniqueness of solutions to a backward stochastic differential equation (BSDE) have been extensively investigated in many, but also various specifically chosen settings, partly due to certain applications in practice and partly also for theoretically interesting reasons. In this paper, we both unify and simplify the approach for a general BSDE framework driven by a Lévy process with a straightforward extension to more general filtrations. We show new comparison results and relax the assumptions known so far for guaranteeing unique $L^p$-solutions, $p > 1$, to a BSDE with terminal condition $\xi$ and generator *f* that satisfies a monotonicity condition. An $L^p$-solution is a triplet of processes (*Y*, *Z*, *U*) from suitable $L^p$-spaces (defined in Sect. 2) which satisfies a.s.

1 $$\begin{aligned} Y_t=\xi +\int _t^T f(s,Y_s,Z_s,U_s)ds-\int _t^T Z_s dW_s-\int _{{]t,T]}\times {\mathbb {R}}^d{\setminus }\{0\}}U_s(x){\tilde{N}}(ds,dx), \end{aligned}$$

for each $t\in {[0,T]}$, where *W* is a Brownian motion and ${\tilde{N}}$ is a compensated Poisson random measure independent of *W*. The BSDE (1) itself will be denoted by $(\xi ,f)$.

### Related Works

For nonlinear BSDEs ($\xi$, *f*) driven by Brownian motion, existence and uniqueness results were first systematically studied by Pardoux and Peng [21] with $(\omega ,y,z)\mapsto f(\omega ,y,z)$ Lipschitz in (*z*, *y*) and $\xi$ square integrable. The importance of BSDEs in mathematical finance and stochastic optimal control was further elaborated by various works, e.g., by El Karoui et al. [7] which consider Lipschitz generators, $L^p$-solutions and Malliavin derivatives of BSDEs in the Brownian setting. The ambition to weaken the assumptions on *f* and $\xi$ to still guarantee a unique solution gave birth to a large number of contributions, where—in the case of a generator with Lipschitz dependence on the *z*-variable—at least a few should be mentioned herein: Pardoux [20] and Briand and Carmona [2] considered monotonic generators w.r.t. *y* with different growth conditions. Mao [15] used the Bihari–LaSalle inequality to generalize the growth condition. Briand et al. [3] proved existence and uniqueness of a solution in the case where the generator may have a general growth in the *y*-variable and both $\int _0^T |f(s,0,0)|ds$ and $\xi$ belong to $L^p$ for some $p \ge 1$. Generalizing the driving randomness, Tang and Li [28] and many other papers studied BSDEs including jumps by a Poisson random measure independent of the Brownian motion. Treating BSDEs in the case of quadratic growth in the *z*-variable, a considerable amount of articles was published in the recent years starting from the seminal paper of Kobylanski [12] in 2000 to recent papers using BMO methods such as [4] in the Brownian case or also comparison theorems like in [9] who consider an additional Poisson random measure as driving noise. We skip detailed comments in the direction of quadratic growth BSDEs as we will not consider this setting in our article.

Recent and most relevant for the present paper are the results by Kruse and Popier [13] considering $L^p$-solutions for BSDEs driven by Brownian motion, a Poisson random measure and an additional martingale term under a monotonicity condition. They included the case of random time horizons. Yao [29] studied $L^p$-solutions to BSDEs with a finite activity Lévy process for $1< p < 2$ and used a generalization for the monotonicity assumption similar to the one of [15] and also used in Sow [26]. Generalizing the $L^p$-assumptions for the monotonic generator setting, in [8] the existence (and uniqueness in [5]) of a solution was proved for a scalar linearly growing BSDE when the terminal value $\xi$ admitted integrability of $|\xi |\exp \left( \mu \sqrt{2\log (1+|\xi |)}\right)$ for a parameter $\mu > \mu _0$, for some critical value $\mu _0 > 0$. Moreover, a counterexample in [8] shows that for the case $\mu < \mu _0$ the preceding integrability is not sufficient to guarantee existence. In the critical case $\mu =\mu _0$, they prove existence and uniqueness of a solution assuming a uniform Lipschitz generator.

### Main Contribution

Within our approach, the results shed new light on the extensive literature of BSDE existence and uniqueness results as follows. In [14], Kruse and Popier designed function spaces such that their results of [13] extend to $1<p<2$. In the present article, we show that the BSDEs’ solutions for $1<p<2$ are even contained in the usual $L^p$ spaces as defined for $p\ge 2$. Moreover, an additional martingale term *M* orthogonal to *W* and ${\tilde{N}}$ as used by Kruse and Popier [13] can also be added to our setting as an extension of (1),

2 $$\begin{aligned} Y_t&=\xi +\int _t^T f(s,Y_s,Z_s,U_s)ds-\int _t^T Z_s dW_s \nonumber \\&\quad -\int _{{]t,T]}\times {\mathbb {R}}^\ell {\setminus }\{0\}}U_s(x){\tilde{N}}(ds,dx) - \int _t^T dM_s, \end{aligned}$$

with unknown variables (*Y*, *Z*, *U*, *M*), as the careful analysis in their paper shows how the bracket process [*M*] has to be treated in an a priori estimate. All the results we obtain are still valid in this extended setting—see Remark 1. Nonetheless, we decided to omit the presentation of the straightforward martingale term, since the main difficulty lies in the treatment of the compensated Poisson random measure.

The paper of Geiss and Steinicke [10], placed in a one-dimensional $L^2$-setting only, requires a linear growth condition on the generator and needs approximation results for the comparison theorem, while the present setting allows first of all general growth, but even uses a simpler approximation technique for the comparison theorem avoiding deep-lying measurability results and, for $p\ge 2$, only requires comparison of the generators on the solution processes.

Furthermore, in contrast to [3, 13] and others, this article establishes the more general monotonicity (Osgood) condition with a nondecreasing, concave function $\rho$ to relax the generator’s dependence on *y* (see also Mao [15]). This includes, e.g., continuities of the type as the function $y\mapsto -y\log (|y|)$ possesses at $y=0$. Using the general approach, similar a priori estimates are shown to still hold true in order to guarantee uniqueness of an $L^p$-solution, $p\ge 2$.

In addition, the results of Yao [29] are extended in the sense that we do not require the jump process to have a finite Lévy measure.

Hence, we close several gaps in the theoretical understanding of solutions to BSDEs driven by a Lévy process, for the class of generators which are Lipschitz in the *z*- and *u*-variables. The more delicate techniques needed for this paper’s approach for existence and uniqueness are inspired by the ideas of [3] along with [13] and [7]. In that spirit, before starting the main proofs, we obtain useful a priori estimates for the solution processes. For the comparison theorem, we enhance ideas and simplify proofs from [10] and [23].

### Structure of the Paper

This paper is organized in the following way: First we establish the setting in Sect. 2 and state the assumptions and the main theorem (Sect. 3). After developing a priori estimates in Sect. 4, we finally prove existence and uniqueness of $L^p$-solutions for $p > 1$ in Sect. 5 and end up with the comparison results for $p \ge 2$ and $1< p < 2$ in Sect. 6.

## Setting

Throughout the paper, we will use the following setting: In dimension $d \ge 1$, let $|\cdot |$ denote the Euclidean distance. For $x,y \in {\mathbb {R}}^d$, we write ${\langle x , y \rangle } = \sum _{i=1}^d x_i y_i$, and for $z \in {\mathbb {R}}^{d \times k}, k\ge 1,$ we denote $|z|^2 = {{\,\mathrm{trace}\,}}({z z^*})$. The operations $\min (a,b)$ and $\max (a,b)$ will be denoted by $a \wedge b$ and $a \vee b$.

Let $X=\left( X_t\right) _{t\in {[0,T]}}$ be a càdlàg Lévy process with values in ${\mathbb {R}}^d$ on a complete probability space $({\varOmega },{{\mathscr {F}}},{\mathbb {P}})$ with Lévy measure $\nu$. By $\left( {{{\mathscr {F}}}_t}\right) _{t\in {[0,T]}}$, we will denote the augmented natural filtration of *X* and assume that ${{\mathscr {F}}}={{\mathscr {F}}}_T.$ Equations or inequalities for objects on these spaces are considered up to ${\mathbb {P}}$-null sets. Conditional expectations ${\mathbb {E}}\left[ \ \cdot \ \big |{{\mathscr {F}}}_t\right]$ will be denoted by ${\mathbb {E}}_t$.

The Lévy–Itô decomposition of *X* can be written as

3 $$\begin{aligned} X_t = a t + {\varSigma }W_t + \int _{{]0,t]}\times \{ |x|\le 1\}} x{\tilde{N}}(ds,dx) + \int _{{]0,t]}\times \{ |x|> 1\}} x N(ds,dx), \end{aligned}$$

where $a\in {\mathbb {R}}^d$, ${\varSigma }\in {\mathbb {R}}^{d \times k}$ with full column rank, *W* is a *k*-dimensional standard Brownian motion and *N* (${{\tilde{N}}}$) is the (compensated) Poisson random measure corresponding to *X*. For the general theory of Lévy processes, we refer to [1] or [24]. This setting can be adapted to a pure jump process, if one sets ${\varSigma }= 0$ and omits the stochastic integrals with respect to *W* in the BSDE. Generalizing the above setting slightly, ${{\mathscr {F}}}$ can be assumed to be generated by a *k*-dimensional Brownian motion *W* and an independent (from *W*) compensated Poisson random measure ${\tilde{N}}$ on ${\mathbb {R}}^\ell {\setminus }\{0\}$ for some $\ell \ge 1$, and the assumption of a driving process *X* can in principle be omitted. For convenience, however, we will stick to the setting emerging from a driving Lévy process *X*.

### Notation

Let $$0<p\le \infty $$.

- We use the notation $(L^p,\Vert \cdot \Vert _p):=\left( L^p({\varOmega },{{\mathscr {F}}},{\mathbb {P}}),\Vert \cdot \Vert _{L^p}\right)$ for the space of all ${{\mathscr {F}}}$-measurable functions $g : {\varOmega }\rightarrow {\mathbb {R}}^d$ with
  $$\begin{aligned}&||g||_{L^p} := \left( \int _{{\varOmega }} |g|^p d{\mathbb {P}}\right) ^{1/p}< \infty \, \,\,\, \text {if } p< \infty , \,\,\, \text { and } \\&||g||_{L^\infty } := {{\,\mathrm{esssup}\,}}_{\omega \in {\varOmega }} |g(\omega )| < \infty . \end{aligned}$$
- Let ${{\mathscr {S}}}^p$ denote the space of all $({{\mathscr {F}}}_t)_{t \in [0,T]}$-progressively measurable and càdlàg processes $Y:{\varOmega }\times {[0,T]} \rightarrow {\mathbb {R}}^d$ such that
  $$\begin{aligned} \left\| Y\right\| _{{{\mathscr {S}}}^p}:=\Big \Vert \sup _{0\le t\le T} \left| Y_{t}\right| \Big \Vert _p < \infty . \end{aligned}$$
- We define $$L^p(W) $$ as the space of all progressively measurable processes $$Z:{\varOmega }\times {[0,T]}\rightarrow {\mathbb {R}}^{d \times k}$$ such that
  $$\begin{aligned} \left\| Z\right\| _{L^p(W) }:=\left\| \left( \int _0^T\left| Z_s\right| ^2 ds\right) ^\frac{1}{2}\right\| _p < \infty . \end{aligned}$$
- Let ${\mathbb {R}}_0^d:= {\mathbb {R}}^d{\setminus }\{0\}$. We define $L^p({{\tilde{N}}})$ as the space of all random fields $U:{\varOmega }\times {[0,T]}\times {{\mathbb {R}}_0^d}\rightarrow {\mathbb {R}}^d$ which are measurable with respect to ${{\mathscr {P}}}\otimes {{\mathscr {B}}}({\mathbb {R}}_0^d)$ (where ${{\mathscr {P}}}$ denotes the predictable $\sigma$-algebra on ${\varOmega }\times [0,T]$ generated by the left continuous $({{\mathscr {F}}}_t)_{t \in [0,T]}$-adapted processes and ${{\mathscr {B}}}$ is the Borel-$\sigma$-algebra) such that
  $$\begin{aligned} \left\| U\right\| _{L^p({{\tilde{N}}}) }:=\left\| \left( \int _0^T\int _{{\mathbb {R}}_0^d}\left| U_s(x)\right| ^2 \nu (dx)ds\right) ^\frac{1}{2}\right\| _p < \infty . \end{aligned}$$
- $$L^2(\nu ):= L^2({\mathbb {R}}_0^d, {{\mathscr {B}}}({\mathbb {R}}_0^d), \nu ),$$ $$\Vert \cdot \Vert :=\Vert \cdot \Vert _{L^2(\nu )}.$$
- $L^p([0,T]):=L^p([0,T],{{\mathscr {B}}}([0,T]), \lambda )$, where $\lambda$ is the Lebesgue measure on [0, *T*].
- $L_{loc}(W)$ denotes the space of ${\mathbb {R}}^{d \times k}$-valued progressively measurable processes, such that for every $t > 0$,
  $$\begin{aligned} \int _0^t | Z_s |^2 ds < \infty , \quad {\mathbb {P}}\text {-a.s.} \end{aligned}$$
- $L_{loc}({\tilde{N}})$ denotes the space of ${{\mathscr {P}}}\otimes {{\mathscr {B}}}({\mathbb {R}}_0^d)$-measurable random fields $U:{\varOmega }\times {[0,T]}\times {{\mathbb {R}}_0^d}\rightarrow {\mathbb {R}}^d$, such that for every $t > 0$,
  $$\begin{aligned} \int _0^t\int _{{\mathbb {R}}_0^d} \left( | U_s(x) |^2\vee |U_s(x)|\right) \nu (dx)ds < \infty , \quad {\mathbb {P}}\text {-a.s.} \end{aligned}$$
- With a slight abuse of notation, we define
  $$\begin{aligned}&L^p({\varOmega }; L^1([0,T])) \\&\quad := \left\{ F : {\varOmega }\times [0, T] \rightarrow {\mathbb {R}}: F \text { is } {{\mathscr {F}}}\otimes {{\mathscr {B}}}([0,T])\text {-measurable, } { \left\| \int _0^T |F(\omega , t)| dt\right\| _p }< \infty \right\} . \end{aligned}$$
  For $F \in L^p({\varOmega }; L_1([0,T]))$, we define
  4 $$\begin{aligned} I_F(\omega ):= \int _0^T F(\omega , t)dt \quad \text { and } \quad K_F(\omega , s) := \Bigg \{\begin{array}{lr} \frac{F(\omega , s)}{I_F(\omega )}, &{} \text {if } I_F(\omega ) \ne 0\\ 0, &{} \text {if } I_F(\omega ) = 0 \end{array}. \end{aligned}$$
  The notions $I_F$ and $K_F$ are designed to make use of the simple properties, $F = I_F K_F$ and $I_F^{p-1} \int _0^T F(t) dt = I_F^p$ together with $\int _0^T K_F dt = 1$, ${\mathbb {P}}$-a.s. These properties are used, e.g., equation (15).
- We consider the terminal condition $\xi$ to be an ${{\mathscr {F}}}_T$-measurable random variable and the generator to be a random function $f: {\varOmega }\times [0,T] \times {\mathbb {R}}^d\times {\mathbb {R}}^{d \times k}\times L^2(\nu ) \rightarrow {\mathbb {R}}^d$.

#### Definition 1

An $L_{loc}$**-solution to a BSDE** $(\xi ,f)$ with terminal condition $\xi$ and generator *f* is a triplet

$$\begin{aligned} (Y,Z,U)\in L_{loc}(W)\times L_{loc}(W)\times L_{loc}({{\tilde{N}}}), \end{aligned}$$

adapted to $\left( {{{\mathscr {F}}}_t}\right) _{t\in {[0,T]}}$, which satisfies for all $t\in {[0,T]}$,

$$\begin{aligned} Y_t=\xi +\int _t^T f(s,Y_s,Z_s,U_s)ds-\int _t^T Z_s dW_s-\int _{{]t,T]}\times {\mathbb {R}}_0^d}U_s(x){\tilde{N}}(ds,dx),\quad {\mathbb {P}}\text {-a.s}. \end{aligned}$$

#### Definition 2

An $L^p$**-solution to a BSDE** $(\xi ,f)$ with terminal condition $\xi$ and generator *f* is an $L_{loc}$-solution (*Y*, *Z*, *U*) to the BSDE $(\xi ,f)$ which satisfies

$$\begin{aligned} (Y,Z,U)\in {{\mathscr {S}}}^p\times L^p(W)\times L^p({{\tilde{N}}}). \end{aligned}$$

#### Remark 1

An extension of our setting in the way of [14] is the following:

Redefine the above spaces using a filtration $\left( {{{\mathscr {F}}}_t}\right) _{t\in {[0,T]}}$ on $({\varOmega }, {{\mathscr {F}}}, {\mathbb {P}})$, which is assumed to be quasi-left continuous, satisfies the usual conditions and supports a *k*-dimensional Brownian motion *W* and a compensated Poisson random measure ${\tilde{N}}$ on ${\mathbb {R}}^\ell {\setminus }\{0\}$. Furthermore, we introduce the space ${{\mathscr {M}}}_{loc}$ of càdlàg local martingales orthogonal to *W* and ${\tilde{N}}$ and the space ${{\mathscr {M}}}$ of true martingale processes in ${{\mathscr {M}}}_{loc}$. Moreover, define

$$\begin{aligned} {{\mathscr {M}}}^p := \left\{ M \in {{\mathscr {M}}} : {\mathbb {E}}\left[ ([M]_T)^{p/2} \right] < \infty \right\} , \end{aligned}$$

and in the sense of Definition 1 and 2, let an $L_{loc}$-solution (respectively, $L^p$-solution) to a BSDE (2) be a tuple

$$\begin{aligned} (Y,Z,U,M)\in L_{loc}(W)\times L_{loc}(W)\times L_{loc}({{\tilde{N}}}) \times {{\mathscr {M}}}_{loc}, \end{aligned}$$

respectively,

$$\begin{aligned} (Y,Z,U,M)\in {{\mathscr {S}}}^p\times L^p(W)\times L^p({{\tilde{N}}}) \times {{\mathscr {M}}}^p \end{aligned}$$

satisfying equation (2) instead of (1). As mentioned in the introduction, our main results in the following sections can also be shown within the extended setting producing some extra lines of technical computations.

### Lévy Process with Finite Measure

The driving Lévy process, given by its Lévy–Itô decomposition (3), will be approximated for $n\ge 1$ by

$$\begin{aligned}X^n_t=at+{\varSigma }W_t+\int _{]0,t]\times \{|x|>1\}}x N(ds,dx)+\int _{]0,t]\times \{1/n \le |x|\le 1\}}x {\tilde{N}}(ds,dx).\end{aligned}$$

The process $X^n$ has a finite Lévy measure. Note furthermore that the compensated Poisson random measure associated with $X^n$ can be expressed as ${\tilde{N}}^n=\chi _{\{1/n \le |x|\}}{\tilde{N}}$, where $\chi _A$ denotes the indicator function of a set *A*. Let

$$\begin{aligned} {{\mathscr {F}}}^0&:=\{{\varOmega }, \emptyset \} \vee {{\mathscr {N}}}, \\ {{\mathscr {F}}}^n&:= \sigma (X^n) \vee {{\mathscr {N}}}, \quad n\ge 1, \end{aligned}$$

where ${{\mathscr {N}}}$ stands for the null sets of ${{\mathscr {F}}}.$ Denote by ${\mathbb {E}}_n$ the conditional expectation ${\mathbb {E}}\left[ \ \cdot \ \big |{{\mathscr {F}}}^n\right]$.

## Main Theorem

With this setting in mind, we now state the main theorem based on the following assumptions, with a slight distinction for $p \ge 2$ and $p < 2$, which turns out to be quite natural for the proofs. Instead of a Lipschitz condition, we require the weaker conditions (${\text {A3}}_{\ge 2}$) and, respectively (${\text {A3}}_{< 2}$), referred to as one-sided Lipschitz or monotonicity condition for the generator *f*.

### Assumptions

(A 1): For all $(y,z,u) \in {\mathbb {R}}^d\times {\mathbb {R}}^{d \times k}\times L^2(\nu ): (\omega ,s)\mapsto f(\omega ,s,y,z,u)$ is progressively measurable and the process $f_0=(f(t,0,0,0))_{t\in {[0,T]}}$ is in $L^p({\varOmega };L^1([0,T]))$.
(A 1): For all $r>0$, there are nonnegative, progressively measurable processes ${\varPhi }$, $\psi _r$ with
  $$\begin{aligned} \left\| \int _0^T {\varPhi }(\cdot ,s)^2ds \right\| _{\infty }<\infty \end{aligned}$$
  and $\psi _r\in L^1({\varOmega }\times {[0,T]})$ such that for all $(z,u) \in {\mathbb {R}}^{d \times k}\times L^2(\nu )$,
  $$\begin{aligned}&\sup _{|y|\le r}|f(t,y,z,u)-f_0(t)|\le \psi _r(t)+{\varPhi }(t)(|z|+\Vert u\Vert ), \quad {\mathbb {P}}\otimes \lambda \text {-a.e.} \end{aligned}$$
($${\text {A3}}_{\ge 2}$$)
For $$p\ge 2$$:
  For $\lambda$-almost all *s*, the mapping $(y,z,u)\mapsto f(s,y,z,u)$ is ${\mathbb {P}}$-a.s. continuous. Moreover, there is a nonnegative function $\alpha \in L^1([0,T])$ and progressively measurable processes $\mu , \beta$ with $\int _0^T\left( \mu (\omega ,s)+\beta (\omega ,s)^2\right) ds < \infty$, ${\mathbb {P}}$-a.s. such that for all $(y,z,u), (y',z',u') \in {\mathbb {R}}^d\times {\mathbb {R}}^{d \times k}\times L^2(\nu )$,
  5 $$\begin{aligned}&|y-y'|^{p-2} {\langle y-y' , f(t,y,z,u)-f(t,y',z',u') \rangle } \nonumber \\&\quad \le \alpha (t)|y-y'|^{p-2}\rho (|y-y'|^2)+\mu (t)|y-y'|^p \nonumber \\&\qquad +\beta (t)|y-y'|^{p-1}(|z-z'|+\Vert u-u'\Vert ), \end{aligned}$$
  ${\mathbb {P}}\otimes \lambda \text {-a.e.}$, for a nondecreasing, continuous and concave function $\rho$ from ${[0,\infty [}$ to itself, satisfying $\rho (0)=0$, $\lim _{x\rightarrow 0} \frac{\rho (x^2)}{x} = 0$ and the Osgood condition $\int _{0^+}\frac{1}{\rho (x)}dx := \int _{0}^\epsilon \frac{1}{\rho (x)}dx=\infty$, for some $\epsilon > 0$.
($${\text {A3}}_{< 2}$$)
For $$0<p<2$$:
  For $\lambda$-almost all *s*, the mapping $(y,z,u)\mapsto f(s,y,z,u)$ is ${\mathbb {P}}$-a.s. continuous. Moreover, there is a nonnegative function $\alpha \in L^1([0,T])$, $C>0$ and progressively measurable processes $\mu , \beta _1, \beta _2$ with $\int _0^T\left( \mu (\omega ,s)+\beta _1(\omega ,s)^2+\beta _2(\omega ,s)^q\right) ds < C$, ${\mathbb {P}}$-a.s for some $q > 2$. such that for all $(y,z,u), (y',z',u') \in {\mathbb {R}}^d\times {\mathbb {R}}^{d \times k}\times L^2(\nu )$, $y\ne y'$
  6 $$\begin{aligned}&|y-y'|^{p-2}{\langle y-y' , f(t,y,z,u)-f(t,y',z',u') \rangle } \nonumber \\&\quad \le \alpha (t)\rho (|y-y'|^p)+\mu (t)|y-y'|^p \nonumber \\&\qquad +|y-y'|^{p-1} \left( \beta _1(t)|z-z'|+\beta _2(t)\Vert u-u'\Vert \right) , \end{aligned}$$
  ${\mathbb {P}}\otimes \lambda \text {-a.e.}$, for a nondecreasing, continuous and concave function $\rho$ from ${[0,\infty [}$ to itself, satisfying $\rho (0)=0$, $\lim _{x \rightarrow 0} \frac{\rho (x^p)}{x^{p-1}} = 0$ and $\int _{0^+}\frac{1}{\rho (x)}dx=\infty$.

#### Remark 2

(i) The limit assumptions $\lim _{x \rightarrow 0} \frac{\rho (x^2)}{x} = 0$ together with (5) or $\lim _{x \rightarrow 0} \frac{\rho (x^p)}{x^{p-1}} = 0$ together with (6) already imply that the generator *f* is Lipschitz in *z*, *u*. Moreover, $\beta$ (and analogously for (${\text {A3}}_{< 2}$) the process $\beta _1 + \beta _2$) can take the role of ${\varPhi }$ in (A 2). Nonetheless, for convenience in the proofs, we will still use the generic function ${\varPhi }$.
(ii) The $\rho$-function appearing in the right-hand sides of (${\text {A3}}_{\ge 2}$) and (${\text {A3}}_{< 2}$) admits the following inequalities, which play important roles in the proofs:
  - $$\rho (|y|^2)|y|^{p-2} \le \rho (|y|^p) + \rho (1) |y|^p, \quad \text {for } p \ge 2$$,
  - $$\rho (|y|^p)|y|^{2-p} \le \rho (|y|^2) + \rho (1) |y|^2, \quad \text {for } 0< p < 2$$.

#### Proof

For (ii), we see that, if $|y| < 1$, then $|y|^{p-2} < 1$ and by the concavity of $\rho$,

$$\begin{aligned} \rho (|y|^2)|y|^{p-2} \le \rho (|y|^2|y|^{p-2}) = \rho (|y|^p). \end{aligned}$$

For $|y| \ge 1$, we have by the concavity of $\rho$,

$$\begin{aligned} \rho (|y|^2)|y|^{p-2} \le \rho (1)|y|^2|y|^{p-2} = \rho (1)|y|^{p}. \end{aligned}$$

The case $0 < p \le 2$ is similar. $\square$

#### Remark 3

(i) In (${\text {A3}}_{< 2}$), if $\beta _2$ is deterministic, we could impose the weaker condition $q=2$ as described later in Remark 5.
(ii) The following example is constructed in order to demonstrate the possibilities in this setting for $d=1, p>1$. All the involved expressions are chosen to exploit the assumptions on the coefficients which may be time-dependent, unbounded and some even random. The generator’s dependence on *y* is not Lipschitz (not even one-sided Lipschitz) and of super-linear growth:
  $$\begin{aligned}&f(\omega ,t,y,z,u) \\&\quad =\frac{-1}{\sqrt{t}}y\log (|y|)-\mu (\omega ,t)\left( y^3+y^\frac{1}{3}\right) +\beta _1(\omega ,t)(z+\sin (z)\cos (y))\\&\qquad +\beta _2(\omega ,t)\int _{{\mathbb {R}}_0}\left( \arctan (y\kappa (x)u(x))+u(x)\right) \kappa (x)\nu (dx)+f_0(t), \end{aligned}$$
  where
  $\mu$ is given by $\mu (\omega ,t)=\sum _{n=1}^\infty \frac{1}{n^2\sqrt{t-t_n(\omega )}}$, with $(t_n(\omega ))_{n\ge 1}$ being a numeration of the jumps of the trajectory $t\mapsto X_t(\omega )$ of the Lévy process and $\mu (t,\omega )=0$ if $t\mapsto X_{t}(\omega )$ has no jumps,
  $$\beta _1(\omega ,t)={\left\{ \begin{array}{ll}\frac{\chi _{[T/2,T]}(t)}{\sqrt{|t-W_{T/2}(\omega )|}\left( |\log (|t-W_{T/2}(\omega )|+1)|\right) },&{} \text {when defined,}\\ \quad \quad \quad \quad 0 &{} \text {else,}\end{array}\right. }$$
  $$\beta _2(\omega ,t)={\left\{ \begin{array}{ll}\frac{\chi _{[T/3,T]}(t)}{\left| \log \left( \left| t-\frac{|W_{T/3}(\omega )|}{1+|W_{T/3}(\omega )|}\right| \right) \right| },&{} \text {when defined,}\\ \quad \quad \quad \quad 0 &{} \text {else,}\end{array}\right. }$$
  $$\kappa (x)=1\wedge |x|$$,
  $$f_0(\omega ,t)={\left\{ \begin{array}{ll} \left( \int _0^t\frac{\sqrt{s}\ \exp \left( \frac{W_s^2}{2s}\right) }{|W_s|\left( |\log \left( \frac{|W_s|}{\sqrt{t}}\right) |+1\right) ^2}ds\right) ^\frac{1-p}{p}\frac{\sqrt{t}\ \exp \left( \frac{W_t^2}{2t} \right) }{|W_t|\left( |\log \left( \frac{|W_t|}{\sqrt{t}}\right) |+1\right) ^2}, &{} \text {when defined,}\\ \quad \quad \quad \quad 0 &{} \text {else.}\end{array}\right. }$$

### Main Theorem

#### Theorem 1

(Existence and uniqueness) Assume that the terminal condition $\xi$ is in $L^p$ and the generator *f* satisfies (A 1),(A 2),(A$3\ge 2$), for $p\ge 2$ or (A 1), (A 2), (A$3<2$), for $1<p<2$, then there exists a unique $L^p$-solution to the BSDE $(\xi ,f)$.

We will prove this theorem in Sect. 5 after presenting necessary a priori estimates in the next section.

## A Priori Estimates and Stability

Throughout the next sections, recall that $f_0(t)= f(t,0,0,0)$, and that $I_{|f_0|}$ and $K_{|f_0|}$ are defined as in (4).

### Remark 4

For the results in this section, it suffices to require a weaker condition than (${\text {A3}}_{\ge 2}$). We define this adapted assumption (${\text {a3}}_{< 2}$) with the same requirements, except one: We replace the monotonicity condition (5) by

($${\text {a3}}_{\ge 2}$$)
  7 $$\begin{aligned}&|y|^{p-2} {\langle y , f(t,y,z,u) \rangle } \nonumber \\&\quad \le \alpha (t)|y|^{p-2}\rho (|y|^2)+\mu (t)|y|^p+\beta (t)|y|^{p-1}(|z|+\Vert u\Vert )+|y|^{p-1}|f_0(t)|, \end{aligned}$$
  and analogously, we adapt the assumption (${\text {A3}}_{< 2}$) to derive the weaker (${\text {a3}}_{< 2}$) by replacing inequality (6),
($${\text {a3}}_{< 2}$$)
  8 $$\begin{aligned}&|y|^{p-2} {\langle y , f(t,y,z,u) \rangle } \nonumber \\&\quad \le \alpha (t)\rho (|y|^p)+\mu (t)|y|^p+|y|^{p-1}\left( \beta _1(t)|z|+\beta _2(t)\Vert u\Vert \right) +|y|^{p-1}|f_0(t)|, \end{aligned}$$
  $${\mathbb {P}}\otimes \lambda \text {-a.e.}$$ for all $$(y,z,u) \in {\mathbb {R}}^d\times {\mathbb {R}}^{d \times k}\times L^2(\nu )$$.

### Lemma 1

If a sequence of random variables $(V_n)_{n \in {\mathbb {N}}}$ in $L^p$ satisfies $\lim _{n\rightarrow \infty }{\mathbb {E}}|V_n|^p=0$, then for a function $\rho$ as in the assumptions, we have

$$\begin{aligned} \lim _{n\rightarrow \infty }{\mathbb {E}}\left[ \rho \left( |V_n|^2\right) ^{\frac{p}{2}}\right] = 0. \end{aligned}$$

### Proof

This follows from the continuity of $\rho$, $\rho (0) = 0$ and the uniform integrability of $(|V_n|^p)_{n\ge 1}$,

$$\begin{aligned} \rho \left( |V_n|^2\right) ^{\frac{p}{2}} \le \left( a + b |V_n|^2\right) ^{\frac{p}{2}} \le 2^{\frac{p}{2}-1} \left( a^{\frac{p}{2}} + b^{\frac{p}{2}} |V_n|^p\right) , \end{aligned}$$

since $\rho (x)\le a + bx$ for some $a,b>0$ and the above inequality shows that also $(\rho (|V_n|^2)^{\frac{p}{2}})_{n\ge 1}$ is a uniformly integrable sequence. $\square$

The following two propositions show that the norms of the *Z* and *U* processes can be controlled by expressions in *Y* and $f_0$. Note that the bounds in Proposition 1 and Proposition 2 differ slightly, so that the application of Proposition 1 in Sect. 5 needs the assertion of Lemma 1.

### Proposition 1

Let $p \ge 2$ and let (*Y*, *Z*, *U*) be an $L_{loc}$-solution to the BSDE $(\xi ,f)$. If $\xi \in L^p$, $Y \in {{\mathscr {S}}}^p$ and (A 1) and (${\text {a3}}_{\ge 2}$) are satisfied, then (*Y*, *Z*, *U*) is an $L^p$-solution.

More precisely, there is a constant $C > 0$ depending on $p,T,\alpha ,\mu ,\beta$ such that for all $t\in {[0,T]}$,

$$\begin{aligned}&{\mathbb {E}}\left[ \left( \int _t^{T}|Z_s|^2ds\right) ^\frac{p}{2}\right] +{\mathbb {E}}\left[ \left( \int _t^{T}\Vert U_s\Vert ^2 ds\right) ^\frac{p}{2}\right] \\&\quad \le C\left( {\mathbb {E}}\left[ \sup _{s\in {[t,T]}}|Y_s|^p\right] + {\mathbb {E}}\left[ \rho \left( \sup _{s\in {[t,T]}}|Y_s|^2\right) ^{\frac{p}{2}}\right] +{\mathbb {E}}\left[ \left( \int _t^T|f_0(s)|ds\right) ^p\right] \right) . \end{aligned}$$

### Proof

This proof generalizes the arguments in [3, Lemma 3.1].

**Step 1:**

For $t\in {[0,T]}$ and $n \ge 1$, define the stopping times

$$\begin{aligned} \tau _n:=\inf \left\{ s\in [t,T]: \int _t^{s}|Z_s|^2ds\ge n\right\} \wedge \inf \left\{ s\in {[t,T]}: \int _t^{s}\Vert U_s\Vert ^2 ds\ge n\right\} . \end{aligned}$$

Itô’s formula implies

9 $$\begin{aligned}&|Y_t|^2+\int _t^{\tau _n}|Z_s|^2ds+\int _t^{\tau _n}\Vert U_s\Vert ^2 ds\nonumber \\&\quad =|Y_{\tau _n}|^2+2\int _t^{\tau _n} {\langle Y_s , f(s,Y_s,Z_s,U_s) \rangle }ds \nonumber \\&\qquad -2\int _t^{\tau _n} {\langle Y_s , Z_sdW_s \rangle } -\int _{{]t,\tau _n]}\times {\mathbb {R}}_0^d}\left( |Y_{s-}+U_s(x)|^2-|Y_{s-}|^2\right) {\tilde{N}}(ds,dx), \end{aligned}$$

from which we infer by (${\text {a3}}_{\ge 2}$) that

$$\begin{aligned}&\int _t^{\tau _n}|Z_s|^2ds+\int _t^{\tau _n}\Vert U_s\Vert ^2 ds\\&\quad \le |Y_{\tau _n}|^2+2\int _t^{\tau _n}\left( \alpha (s)\rho (|Y_s|^2)+\mu (s)|Y_s|^2\right) ds\\&\qquad +\int _t^{\tau _n}\beta (s)|Y_s|(|Z_s|+\Vert U_s\Vert )ds+2\int _t^{\tau _n}|Y_s||f_0(s)| ds\\&\qquad +2\left| \int _t^{\tau _n} {\langle Y_s , Z_sdW_s \rangle }\right| +\left| \int _{{]t,\tau _n]}\times {\mathbb {R}}_0^d}\left( |Y_{s-}+U_s(x)|^2-|Y_{s-}|^2\right) {\tilde{N}}(ds,dx)\right| . \end{aligned}$$

Taking the power $\frac{p}{2}$, we find a constant $c_0> 0$ such that

$$\begin{aligned}&\left[ \int _t^{\tau _n}|Z_s|^2ds\right] ^\frac{p}{2}+\left[ \int _t^{\tau _n}\Vert U_s\Vert ^2 ds\right] ^\frac{p}{2} \\&\quad \le c_0\biggl (|Y_{\tau _n}|^p+\left[ \int _t^{\tau _n}\left( \alpha (s) \rho (|Y_s|^2)+\mu (s)|Y_s|^2\right) ds\right] ^\frac{p}{2}\\&\qquad +\left[ \int _t^{\tau _n}\beta (s)|Y_s|(|Z_s|+\Vert U_s\Vert )ds \right] ^\frac{p}{2}+\left[ \int _t^{\tau _n}|Y_s||f_0(s)|ds\right] ^\frac{p}{2}\\&\qquad +\left| \int _t^{\tau _n} {\langle Y_s , Z_sdW_s \rangle } \right| ^\frac{p}{2}+\left| \int _{{]t,\tau _n]}\times {\mathbb {R}}_0^d} \left( |Y_{s-}+U_s(x)|^2-|Y_{s-}|^2\right) {\tilde{N}}(ds,dx)\right| ^\frac{p}{2}\biggr ). \end{aligned}$$

We continue our estimate (with another constant $c_1 > 0$)

10 $$\begin{aligned}&\left[ \int _t^{\tau _n}|Z_s|^2ds\right] ^\frac{p}{2}+\left[ \int _t^{\tau _n}\Vert U_s\Vert ^2 ds\right] ^\frac{p}{2} \nonumber \\&\quad \le c_1\Biggl (\sup _{s\in {[t,T]}}|Y_s|^p+\left[ \int _t^T \alpha (s)ds \, \right] ^\frac{p}{2}\rho \left( \sup _{s\in [t,T]}|Y_s|^{2}\right) ^{\frac{p}{2}} +\left[ \int _t^T\mu (s)ds\right] ^\frac{p}{2}\sup _{s\in {[t,T]}}|Y_s|^p \nonumber \\&\qquad +\left[ \int _t^{\tau _n}\beta (s)|Y_s|(|Z_s|+\Vert U_s\Vert )ds\right] ^\frac{p}{2}+\left[ \int _t^{\tau _n}|f_0(s)|ds\right] ^\frac{p}{2}\sup _{s\in {[t,T]}}|Y_s|^\frac{p}{2} \nonumber \\&\qquad +\left| \int _t^{\tau _n} {\langle Y_s , Z_sdW_s \rangle } \right| ^\frac{p}{2}+\left| \int _{{]t,\tau _n]}\times {\mathbb {R}}_0^d}\left( |Y_{s-}+U_s(x)|^2-|Y_{s-}|^2\right) {\tilde{N}}(ds,dx)\right| ^\frac{p}{2}\Biggr ). \end{aligned}$$

To estimate the above further, we have to split up the range of values of $p\ge 2$.

**Case 1:** $$2 \le p \le 4$$

We use the following inequality given, e.g., in [17, Theorem 3.2], which states that for a local martingale *M*, given by $M(t)=\int _{{]0,t]}\times {\mathbb {R}}_0^d} g_s(x){\tilde{N}}(ds,dx), t\in [0,T]$, there exists $c_2> 0$ such that the following inequality holds for $p'\in {]0,2]}$:

$$\begin{aligned} {\mathbb {E}}\sup _{t\in [0,T]}\left| M_t\right| ^{p'}\le c_2{\mathbb {E}}\left[ \left( \int _0^T\int _{{\mathbb {R}}_0^d} |g_s|^2\nu (dx)ds\right) ^\frac{p'}{2}\right] . \end{aligned}$$

Here, we will apply this inequality for $p' = p/2$ to the martingale

$$\begin{aligned} s\mapsto \int _{{]t,s\wedge \tau _n]}\times {\mathbb {R}}_0^d}\left( |Y_{s-}+U_s(x)|^2-|Y_{s-}|^2\right) {\tilde{N}}(ds,dx). \end{aligned}$$

Note that we can estimate the square of the above integrand ${\mathbb {P}}\otimes \lambda \otimes \nu$-a.e. by

11 $$\begin{aligned} \left( |Y_{s-}+ U_s(x)|+|Y_{s-}|\right) ^2 \left( |Y_{s-}+ U_s(x)|-|Y_{s-}|\right) ^2 \le 16 \sup _{r \in [t,T]} |Y_r|^2 \,|U_s(x)|^2, \end{aligned}$$

since for all $s \in [t,T]$ we can bound the jump sizes $|U_s(x)|$ by $2 \sup _{r \in [t,T]} |Y_r|$, ${\mathbb {P}}\otimes \lambda \otimes \nu$-a.e. (see [19, Corollary 1]) and since $\big ||Y_{s-}+ U_s(x)|-|Y_{s-}|\big | \le |U_s(x)|$.

We take suprema and expectations to get a constant $$c_3> 0 $$ such that

$$\begin{aligned}&{\mathbb {E}}\left[ \int _t^{\tau _n}|Z_s|^2ds\right] ^\frac{p}{2}+{\mathbb {E}}\left[ \int _t^{\tau _n}\Vert U_s\Vert ^2 ds\right] ^\frac{p}{2}\\&\quad \le c_3\Biggl ({\mathbb {E}}\left[ \sup _{s\in {[t,T]}}|Y_s|^p\right] +{\mathbb {E}}\left[ \rho \left( \sup _{s\in [t,T]}|Y_s|^{2}\right) ^{\frac{p}{2}}\right] \\&\qquad +{\mathbb {E}}\left[ \int _t^{\tau _n}\beta (s)|Y_s|(|Z_s|+\Vert U_s\Vert )ds\right] ^\frac{p}{2}\\&\qquad +{\mathbb {E}}\left[ \left( \int _t^{\tau _n}|f_0(s)|ds\right) ^\frac{p}{2}\sup _{s\in {[t,T]}}|Y_s|^\frac{p}{2}\right] \\&\qquad +{\mathbb {E}}\left[ \int _t^{\tau _n}|Y_s|^2|Z_s|^2ds\right] ^\frac{p}{4} \\&\qquad +{\mathbb {E}}\left[ \int _t^{\tau _n}\sup _{r\in {[t,T]}}|Y_r|^2\Vert U_s\Vert ^2ds\right] ^\frac{p}{4}\Biggr ). \end{aligned}$$

Young’s inequality (see Theorem 4 in Appendix) now gives us for an arbitrary $R>0$,

$$\begin{aligned}&{\mathbb {E}}\left[ \int _t^{\tau _n}|Z_s|^2ds\right] ^\frac{p}{2}+{\mathbb {E}}\left[ \int _t^{\tau _n}\Vert U_s\Vert ^2 ds\right] ^\frac{p}{2}\\&\quad \le c_3\Biggl ({\mathbb {E}}\left[ \sup _{s\in {[t,T]}}|Y_s|^p\right] +{\mathbb {E}}\left[ \rho \left( \sup _{s\in [t,T]}|Y_s|^{2}\right) ^{\frac{p}{2}}\right] \\&\qquad +{\mathbb {E}}\left[ \int _t^{\tau _n}\frac{R}{2}\beta (s)^2|Y_s|^2ds\right] ^\frac{p}{2} \\&\qquad +\frac{1}{(2R)^\frac{p}{2}}\left( {\mathbb {E}}\left[ \int _t^{\tau _n}|Z_s|^2ds\right] ^\frac{p}{2}+{\mathbb {E}}\left[ \int _t^{\tau _n}\Vert U_s\Vert ^2ds\right] ^\frac{p}{2}\right) \\&\qquad +\frac{1}{2}{\mathbb {E}}\left[ \int _t^{\tau _n}|f_0(s)|ds\right] ^p+\frac{1}{2}{\mathbb {E}}\sup _{s\in {[t,T]}}|Y_s|^p\\&\qquad +2\left( \frac{R}{2}\right) ^\frac{p}{2}{\mathbb {E}}\sup _{s\in {[t,T]}}|Y_s|^p \\&\qquad +\frac{1}{(2R)^\frac{p}{2}}\left( {\mathbb {E}}\left[ \int _t^{\tau _n}|Z_s|^2ds\right] ^\frac{p}{2}+{\mathbb {E}}\left[ \int _t^{\tau _n}\Vert U_s\Vert ^2ds\right] ^\frac{p}{2}\right) \Biggr ). \end{aligned}$$

Choosing now *R* such that $$\frac{2 c_3}{(2R)^\frac{p}{2}}<1$$ yields a constant $$C > 0 $$ such that

$$\begin{aligned}&{\mathbb {E}}\left[ \int _t^{\tau _n}|Z_s|^2ds\right] ^\frac{p}{2}+{\mathbb {E}}\left[ \int _t^{\tau _n}\Vert U_s\Vert ^2 ds\right] ^\frac{p}{2}\\&\quad \le C\left( {\mathbb {E}}\left[ \sup _{s\in {[t,T]}}|Y_s|^p\right] +{\mathbb {E}}\left[ \rho \left( \sup _{s\in [t,T]}|Y_s|^{2}\right) ^{\frac{p}{2}}\right] +{\mathbb {E}}\left[ \int _t^T|f_0(s)|ds\right] ^p\right) . \end{aligned}$$

Taking the limit for $n\rightarrow \infty$ shows the assertion for $2 \le p \le 4$.

**Case 2:** $$p > 4$$

We start from (10) following the same lines of the previous case. In this case, the only difference is: [17, Theorem 3.2] states that for a local martingale *M*, given by $M(t)=\int _{{]0,t]}\times {\mathbb {R}}_0^d} g_s(x){\tilde{N}}(ds,dx)$, $t\in [0,T]$ there exists $c_4> 0$ such that the following inequality holds for all $p' \ge 2$:

12 $$\begin{aligned} {\mathbb {E}}\sup _{t\in [0,T]}\left| M_t\right| ^{p'}\le c_4{\mathbb {E}}\left( \left[ \int _0^T\int _{{\mathbb {R}}_0^d} |g_s|^2\nu (dx)ds\right] ^\frac{p'}{2} + \int _0^T\int _{{\mathbb {R}}_0^d} |g_s|^{p'}\nu (dx)ds\right) . \end{aligned}$$

For $p' = \frac{p}{2}$, we apply this inequality to the local martingale

$$\begin{aligned} s\mapsto \int _{{]t,s\wedge \tau _n]}\times {\mathbb {R}}_0^d}\left( |Y_{s-}+U_s(x)|^2-|Y_{s-}|^2\right) {\tilde{N}}(ds,dx). \end{aligned}$$

The first summand of (12) can be treated as in case 1. We focus on the second term which equals

13 $$\begin{aligned}&\int _t^T\int _{{\mathbb {R}}_0^d} \left( |Y_{s-}+U_s(x)|^2-|Y_{s-}|^2\right) ^{\frac{p}{2}}\nu (dx)ds\nonumber \\&\quad =\int _t^T\int _{{\mathbb {R}}_0^d} \left( |Y_{s-}+U_s(x)|^2-|Y_{s-}|^2\right) ^{\frac{p}{2}-2}\nonumber \\&\qquad \left( |Y_{s-}+U_s(x)|^2-|Y_{s-}|^2\right) ^{2}\nu (dx)ds. \end{aligned}$$

We can bound the integrands (as explained in (11)) by

$$\begin{aligned} \big (|Y_{s-}+ U_s(x)|+|Y_{s-}|\big ) \big (|Y_{s-}+ U_s(x)|-|Y_{s-}|\big ) \le 16 \sup _{r \in [t,T]} |Y_r|^2, \end{aligned}$$

and

$$\begin{aligned} \big (|Y_{s-}+ U_s(x)|+|Y_{s-}|\big ) \big (|Y_{s-}+ U_s(x)|-|Y_{s-}|\big ) \le 4 \sup _{r \in [t,T]} |Y_r| \,|U_s(x)|. \end{aligned}$$

Hence, we find a constant $c_5> 0$, such that (13) is smaller than

$$\begin{aligned} c_5\int _t^T\int _{{\mathbb {R}}_0^d} \sup _{r \in [t,T]} |Y_r|^{p-2} |U_s(x)|^{2} \nu (dx)ds. \end{aligned}$$

Using Young’s inequality for the conjugate couple $(\frac{p}{2}, \frac{p}{p-2})$, we have for arbitrary $R_1 > 0$,

$$\begin{aligned}&\int _t^T\int _{{\mathbb {R}}_0^d} \sup _{r \in [t,T]} |Y_r|^{p-2} |U_s(x)|^{2} \nu (dx)ds \\&\quad = \sup _{r \in [t,T]} |Y_r|^{p-2} \int _t^T\int _{{\mathbb {R}}_0^d} |U_s(x)|^{2} \nu (dx)ds \\&\quad \le \left( \frac{p-2}{p} R_1^{\frac{p}{p-2}} \sup _{r \in [t,T]} |Y_r|^{p} + \frac{2}{pR_1^{\frac{p}{2}}}\left[ \int _t^T\int _{{\mathbb {R}}_0^d} |U_s(x)|^{2} \nu (dx)ds\right] ^{\frac{p}{2}}\right) . \end{aligned}$$

From here, similar steps as in case 1 conclude the proof. $\square$

### Proposition 2

Let $0< p < 2$ and let (*Y*, *Z*, *U*) be an $L_{loc}$-solution to the BSDE $(\xi ,f)$. If $\xi \in L^p$, $Y \in {{\mathscr {S}}}^p$ and (A 1) and (${\text {a3}}_{< 2}$) are satisfied, then (*Y*, *Z*, *U*) is an $L^p$-solution.

More precisely, there is a constant *C* depending on $p,T,\alpha ,\rho (1),\mu ,\beta _1,\beta _2$ such that for all $t\in {[0,T]}$,

$$\begin{aligned}&{\mathbb {E}}\left[ \int _t^{T}|Z_s|^2ds\right] ^\frac{p}{2}+{\mathbb {E}}\left[ \int _t^{T}\Vert U_s\Vert ^2 ds\right] ^\frac{p}{2}\\&\quad \le C\left( {\mathbb {E}}\left[ \sup _{s\in {[t,T]}}|Y_s|^p\right] + {\mathbb {E}}\left[ \int _t^T \alpha (s) \rho \left( |Y_s|^p\right) ds\right] +{\mathbb {E}}\left[ \int _t^T|f_0(s)|ds\right] ^p\right) . \end{aligned}$$

The assertion holds true even if $q=2$ in (${\text {a3}}_{< 2}$) since we do not use a higher integrability condition in the proof.

### Proof

We proceed as in the proof before until (9) and then infer by (${\text {a3}}_{< 2}$) in Remark 4, with $\beta _1+\beta _2=:\beta$, that

$$\begin{aligned}&\int _t^{\tau _n}|Z_s|^2ds+\int _t^{\tau _n}\Vert U_s\Vert ^2 ds\\&\quad \le |Y_{\tau _n}|^2+2\int _t^{\tau _n}\left( \alpha (s)\rho (|Y_s|^p)|Y_s|^{2-p}+\mu (s)|Y_s|^2\right) ds\\&\quad +\int _t^{\tau _n}\beta (s)|Y_s|(|Z_s|+\Vert U_s\Vert )ds+2\int _t^{\tau _n}|Y_s||f_0(s)| ds\\&\quad +2\left| \int _t^{\tau _n} {\langle Y_s , Z_sdW_s \rangle }\right| +\left| \int _{{]t,\tau _n]}\times {\mathbb {R}}_0^d}\left( |Y_{s-}+U_s(x)|^2-|Y_{s-}|^2\right) {\tilde{N}}(ds,dx)\right| . \end{aligned}$$

Taking the power $\frac{p}{2}$, we find a constant $c_0> 0$ such that

$$\begin{aligned}&\left[ \int _t^{\tau _n}|Z_s|^2ds\right] ^\frac{p}{2}+\left[ \int _t^{\tau _n}\Vert U_s\Vert ^2 ds\right] ^\frac{p}{2}\\&\quad \le c_0\Biggl (|Y_{\tau _n}|^p+\left[ \sup _{s \in [t,T]}|Y_s|^{2-p} \int _t^{\tau _n}\alpha (s)\rho (|Y_s|^{p})ds+ \sup _{s \in [t,T]}|Y_s|^2\int _t^{\tau _n}\mu (s)ds\right] ^\frac{p}{2}\\&\qquad +\left[ \int _t^{\tau _n}\beta (s)|Y_s|(|Z_s|+\Vert U_s\Vert )ds\right] ^\frac{p}{2}+\left[ \int _t^{\tau _n}|Y_s||f_0(s)|ds\right] ^\frac{p}{2}\\&\qquad +\left| \int _t^{\tau _n} {\langle Y_s , Z_sdW_s \rangle }\right| ^\frac{p}{2}+\left| \int _{{]t,\tau _n]}\times {\mathbb {R}}_0^d}\left( |Y_{s-}+U_s(x)|^2-|Y_{s-}|^2\right) {\tilde{N}}(ds,dx)\right| ^\frac{p}{2}\Biggr ). \end{aligned}$$

We estimate further with $c_1> 0$

$$\begin{aligned}&\left[ \int _t^{\tau _n}|Z_s|^2ds\right] ^\frac{p}{2}+\left[ \int _t^{\tau _n}\Vert U_s\Vert ^2 ds\right] ^\frac{p}{2} \\&\quad \le c_1\Biggl (|Y_{\tau _n}|^p+\sup _{s \in [t,T]}|Y_s|^{\frac{(2-p)p}{2}} \left[ \int _t^{\tau _n}\alpha (s)\rho (|Y_s|^{p})ds\right] ^{\frac{p}{2}}\\&\qquad + \sup _{s \in [t,T]}|Y_s|^{p}\left[ \int _t^{\tau _n}\mu (s)ds\right] ^\frac{p}{2} +\left[ \int _t^{\tau _n}\beta (s)|Y_s|(|Z_s|+\Vert U_s\Vert )ds\right] ^\frac{p}{2}\\&\qquad +\left[ \int _t^{\tau _n}|Y_s||f_0(s)|ds\right] ^\frac{p}{2} +\left| \int _t^{\tau _n} {\langle Y_s , Z_sdW_s \rangle }\right| ^\frac{p}{2}\\&\qquad +\left| \int _{{]t,\tau _n]}\times {\mathbb {R}}_0^d}\left( |Y_{s-}+U_s(x)|^2 -|Y_{s-}|^2\right) {\tilde{N}}(ds,dx)\right| ^\frac{p}{2}\Biggr ). \end{aligned}$$

With Young’s inequality for $(\frac{2}{p},\frac{2}{2-p})$ and a new constant $c_2 > 0$, we get

$$\begin{aligned}&\left[ \int _t^{\tau _n}|Z_s|^2ds\right] ^\frac{p}{2} +\left[ \int _t^{\tau _n}\Vert U_s\Vert ^2 ds\right] ^\frac{p}{2}\\&\quad \le c_2\Biggl (|Y_{\tau _n}|^p+\sup _{s \in [t,T]}|Y_s|^{p} + \int _t^{\tau _n}\alpha (s)\rho (|Y_s|^{p})ds\\&\qquad + \left[ \int _t^{\tau _n}\beta (s)|Y_s|(|Z_s|+\Vert U_s\Vert )ds\right] ^\frac{p}{2} +\left[ \int _t^{\tau _n}|Y_s||f_0(s)|ds\right] ^\frac{p}{2}\\&\qquad +\left| \int _t^{\tau _n} {\langle Y_s , Z_sdW_s \rangle }\right| ^\frac{p}{2} +\left| \int _{{]t, \tau _n]}\times {\mathbb {R}}_0^d}\left( |Y_{s-}+U_s(x)|^2-|Y_{s-}|^2\right) {\tilde{N}}(ds,dx)\right| ^\frac{p}{2}\Biggr ). \end{aligned}$$

From here on, the proof can be concluded similar to case 1 of Proposition 1. $\square$

From the above proposition, we now know how to bound *Z* and *U* in terms of *Y* and $f_0$. For the core of the existence proof later, we need to control the *Y* part of the solution triplet by a bound depending only on $\xi$ and *f*, which we will show in the sequel.

### Proposition 3

Let $p\ge 2$ and let (*Y*, *Z*, *U*) be an $L^p$-solution to the BSDE $(\xi ,f)$. If $\xi \in L^p$ and (A 1) and (${\text {a3}}_{\ge 2}$) are satisfied, then there exists a function $h:[0,\infty [\rightarrow [0,\infty [$ with $h(x)\rightarrow 0$ as $x\rightarrow 0$ such that

$$\begin{aligned}&\Vert Y\Vert ^p_{{{\mathscr {S}}}^p} \le h\left( {\mathbb {E}}|\xi |^p+{\mathbb {E}}I_{|f_0|}^p\right) , \end{aligned}$$

where *h* depends on $p, T, \rho , \alpha , \beta , \mu$.

### Proof

**Step 1:**

Let ${\varPsi }(y) := |y|^p$ and $\eta$ = $(\eta _t)_{0 \le t \le T} \in L^\infty ({\varOmega }; L^1([0,T]))$ be a progressively measurable, continuous process, which we will determine later. Itô’s formula (see also [13, Proposition 2]) for $t \in [0,T]$ implies

14 $$\begin{aligned}&e^{\int _0^t\eta (\tau )d\tau }|Y_t|^p + \int _t^T e^{\int _0^s\eta (\tau )d\tau }\Big [\eta (s)|Y_s|^p + \frac{1}{2} {{\,\mathrm{trace}\,}}(D^2 {\varPsi }(Y_s)Z_sZ_s^*) \Big ] ds + P(t) \nonumber \\&\quad = e^{\int _0^T\eta (\tau )d\tau }|\xi |^p + \int _t^T e^{\int _0^s\eta (\tau )d\tau }p {\langle Y_s |Y_s|^{p-2} , f(s,Y_s,Z_s,U_s) \rangle } ds + M(t), \end{aligned}$$

where $D^2 {\varPsi }$ denotes the Hessian matrix of ${\varPsi }$,

$$\begin{aligned} P(t) =&\int _t^T \int _{{\mathbb {R}}_0^d} e^{\int _0^s\eta (\tau )d\tau }\\&\left[ |Y_{s-}+U(s,x)|^p - |Y_{s-}|^p - {\langle U(s,x) , p Y_{s-}|Y_{s-}|^{p-2} \rangle }\right] \nu (dx)ds \end{aligned}$$

and

$$\begin{aligned} M(t) =&- \int _t^T e^{\int _0^s\eta (\tau )d\tau }p {\langle Y_{s}|Y_{s}|^{p-2} , Z_s dW_s \rangle } \\&- \int _{]t,T]\times \mathbb {R}_{0}^{d}} e^{\int _0^s\eta (\tau )d\tau }\left[ |Y_{s-}+U(s,x)|^p - |Y_{s-}|^p\right] {\tilde{N}}(ds,dx). \end{aligned}$$

By the argument in [13, Proposition 2], we can use the estimates ${{\,\mathrm{trace}\,}}(D^2 {\varPsi }(y)zz^*) \ge p|y|^{p-2}|z|^2$ and

$$\begin{aligned} P(t) \ge p(1-p)3^{1-p}\int _t^T e^{\int _0^s\eta (\tau )d\tau }|Y_{s-}|^{p-2} \Vert U_s\Vert ^2 ds, \end{aligned}$$

leading to

$$\begin{aligned}&e^{\int _0^t\eta (\tau )d\tau }|Y_t|^p + \int _t^T e^{\int _0^s\eta (\tau )d\tau }\Big [\eta (s)|Y_s|^p + \frac{1}{2} p(p-1)|Y_s|^{p-2} |Z_s|^2 \Big ] ds \\&\qquad +p(1-p)3^{1-p}\int _t^T e^{\int _0^s\eta (\tau )d\tau }|Y_{s}|^{p-2} \Vert U_s\Vert ^2 ds \\&\quad \le e^{\int _0^T\eta (\tau )d\tau }|\xi |^p + \int _t^T e^{\int _0^s\eta (\tau )d\tau }p {\langle Y_s |Y_s|^{p-2} , f(s,Y_s,Z_s,U_s) \rangle }ds + M(t). \end{aligned}$$

Using $c_z = \frac{1}{2}p(1-p)$ and $c_u = p(1-p)3^{1-p}$, (${\text {a3}}_{\ge 2}$), Remark 2(ii)(a), Young’s inequality for arbitrary $R_z, R_u > 0$ with the conjugate couple (2, 2), Young’s inequality once more for the expression ${|Y_s|^{p-1}|f_0| = |Y_s|^{p-1}K_{|f_0|}^{(p-1)/p} |f_0|^{1/p}I_{|f_0|}^{(p-1)/p}}$ (see (4)) and the couple $(\frac{p}{p-1},p)$, we find

15 $$\begin{aligned}&e^{\int _0^t\eta (s)ds}{|Y_t|}^p+\int _t^T e^{\int _0^s\eta (\tau )d\tau }\left( \eta (s){|Y_s|}^p +c_z|Y_s|^{p-2}|Z_s|^2 +c_u{|Y_{s}|^{p-2}}\Vert U_s\Vert ^2\right) ds\nonumber \\&\quad \le e^{\int _0^T\eta (s)ds}|\xi |^p+\int _t^T e^{\int _0^s\eta (\tau )d\tau }p \biggl ( \alpha (s)\rho (|Y_s|^p) \nonumber \\&\qquad +\left( \alpha (s)\rho (1) + \mu (s)+\frac{R_z+R_u}{2}\beta (s)^2\right) |Y_s|^p \biggr )ds \nonumber \\&\qquad +\int _t^T e^{\int _0^s\eta (\tau )d\tau }p|Y_s|^{p-2}\left( \frac{|Z_s|^2}{2R_z}+\frac{\Vert U_s\Vert ^2}{2R_u}\right) ds \nonumber \\&\qquad + \int _t^T e^{\int _0^s\eta (\tau )d\tau } (p-1)|Y_s|^pK_{|f_0|}(s)ds \nonumber \\&\qquad +\int _t^T e^{\int _0^s\eta (\tau )d\tau } |f_0(s)| I_{|f_0|}^{p-1}ds+M(t). \end{aligned}$$

We set $R_z = p/c_z$, $R_u = p/c_u$ and $\eta = p(\alpha \rho (1)+ \mu +\beta ^2(R_z+R_u)/2) + (p-1)K_{|f_0|}$ leading to

$$\begin{aligned}&e^{\int _0^t\eta (s)ds}{|Y_t|}^p + \int _t^T e^{\int _0^s\eta (\tau )d\tau }\left( \frac{c_z}{2}|Y_s|^{p-2}|Z_s|^2 +\frac{c_u}{2}{|Y_{s}|^{p-2}}\Vert U_s\Vert ^2\right) ds \\&\quad \le e^{\int _0^T\eta (s)ds}|\xi |^p+\int _t^T e^{\int _0^s\eta (\tau )d\tau }p \alpha (s)\rho (|Y_s|^p) ds \\&\qquad +\int _t^T e^{\int _0^s\eta (\tau )d\tau } |f_0(s)| I_{|f_0|}^{p-1}ds+M(t). \end{aligned}$$

Now, we omit $e^{\int _0^t\eta (s)ds}{|Y_t|}^p$ and take expectations,

$$\begin{aligned}&{\mathbb {E}}\int _t^T e^{\int _0^s\eta (\tau )d\tau }\left( \frac{c_z}{2}|Y_s|^{p-2}|Z_s|^2 +\frac{c_u}{2}{|Y_{s}|^{p-2}}\Vert U_s\Vert ^2\right) ds \\&\quad \le {\mathbb {E}}e^{\int _0^T\eta (s)ds}|\xi |^p +{\mathbb {E}}\int _t^T e^{\int _0^s\eta (\tau )d\tau }p \alpha (s)\rho (|Y_s|^p) ds \\&\qquad + {\mathbb {E}}I_{|f_0|}^{p-1} \int _t^T e^{\int _0^s\eta (\tau )d\tau } |f_0(s)| ds. \end{aligned}$$

Hence, we find a constant $c_0> 0$, to end the step with

16 $$\begin{aligned}&{\mathbb {E}}\int _t^T \left( |Y_s|^{p-2}|Z_s|^2 +{|Y_{s}|^{p-2}}\Vert U_s\Vert ^2\right) ds \nonumber \\&\quad \le c_0\left( {\mathbb {E}}|\xi |^p +{\mathbb {E}}\int _t^T \alpha (s)\rho (|Y_s|^p) ds + {\mathbb {E}}I_{|f_0|}^{p}. \right) . \end{aligned}$$

**Step 2:**

We take the same route as in the previous step until (15), with one difference: we keep the *P*(*t*) term, to get

17 $$\begin{aligned}&e^{\int _0^t\eta (s)ds}{|Y_t|}^p+\int _t^T e^{\int _0^s\eta (\tau )d\tau }(\eta (s){|Y_s|}^p +c_z|Y_s|^{p-2}|Z_s|^2 )ds \nonumber \\&\quad \le e^{\int _0^T\eta (s)ds}|\xi |^p+\int _t^T e^{\int _0^s\eta (\tau )d\tau }p \nonumber \\&\qquad \biggl ( \alpha (s)\rho (|Y_s|^p) +\left( \alpha (s)\rho (1) + \mu (s)+\frac{R_z+R_u}{2}\beta (s)^2\right) |Y_s|^p \biggr )ds \nonumber \\&\qquad +\int _t^T e^{\int _0^s\eta (\tau )d\tau }p|Y_s|^{p-2}\left( \frac{|Z_s|^2}{2R_z}+\frac{\Vert U_s\Vert ^2}{2R_u}\right) ds \nonumber \\&\qquad + \int _t^T e^{\int _0^s\eta (\tau )d\tau } (p-1)|Y_s|^pK_{|f_0|}(s)ds \nonumber \\&\qquad +\int _t^T e^{\int _0^s\eta (\tau )d\tau } |f_0(s)| I_{|f_0|}^{p-1}ds+M(t) - P(t). \end{aligned}$$

Now we set $R_z = p/(2c_z)$, $R_u = 1/2$ and $\eta = p(\alpha \rho (1)+ \mu +\beta ^2(R_z+R_u)/2) + (p-1)K_{|f_0|}$. By the choice of a suitable constant $c_1> 0$

$$\begin{aligned} e^{\int _0^t\eta (s)ds}{|Y_t|}^p&\le e^{\int _0^T\eta (s)ds}|\xi |^p+\int _t^T e^{\int _0^s\eta (\tau )d\tau }p\left( \alpha (s)\rho (|Y_s|^p) + |Y_s|^{p-2}\Vert U\Vert ^2 \right) ds \\&\quad + c_1I_{|f_0|}^{p} + M(t) - P(t). \end{aligned}$$

We can rewrite

$$\begin{aligned} M(t) - P(t) =&-\int _{t}^{T} e^{\int _0^s\eta (\tau )d\tau }p {\langle Y_{s}|Y_{s}|^{p-2} , Z_s dW_s \rangle } \\&-\int _{{]t, T]}\times {\mathbb {R}}_0^d} e^{\int _0^s\eta (\tau )d\tau }\\&\left[ |Y_{s-}+U(s,x)|^p - |Y_{s-}|^p - {\langle U(s,x) , p Y_{s-}|Y_{s-}|^{p-2} \rangle }\right] N(ds,dx)\\&-\int _{{]t, T]}\times {\mathbb {R}}_0^d} e^{\int _0^s\eta (\tau )d\tau }{\langle U(s,x) , p Y_{s-}|Y_{s-}|^{p-2} \rangle }{\tilde{N}}(ds,dx). \end{aligned}$$

By Taylor expansion of $|\cdot |^p$ (see [13, Proposition 2])

$$\begin{aligned} |Y_{s-}+U(s,x)|^p - |Y_{s-}|^p - {\langle U(s,x) , p Y_{s-}|Y_{s-}|^{p-2} \rangle } \ge 0. \end{aligned}$$

With the minus in front, we can omit the integral with respect to *N*(*ds*, *dx*) in (17), take suprema and end up with

18 $$\begin{aligned} \sup _{s\in {[t,T]}}e^{\int _0^s\eta (\tau )d\tau }{|Y_s|}^p&\le e^{\int _0^T\eta (s)ds}|\xi |^p \nonumber \\&\quad +\int _t^T e^{\int _0^s\eta (\tau )d\tau }p\left( \alpha (s)\rho (|Y_s|^p) + |Y_s|^{p-2}\Vert U\Vert ^2 \right) ds + c_1I_{|f_0|}^{p} \nonumber \\&\quad + \sup _{r\in {[t,T]}} \left| \int _{t}^{r}e^{\int _0^s\eta (\tau )d\tau } p {\langle Y_{s}|Y_{s}|^{p-2} , Z_s dW_s \rangle } \right| \nonumber \\&\quad + \sup _{r\in {[t,T]}} \left| \int _{{]t, \tau _n]}\times {\mathbb {R}}_0^d} e^{\int _0^s\eta (\tau )d\tau }{\langle U(s,x) , p Y_{s-}|Y_{s-}|^{p-2} \rangle }{\tilde{N}}(ds,dx)\right| . \end{aligned}$$

We proceed by estimating the expectation of these two suprema in the next step.

**Step 3:**

For the first supremum, we apply the Burkholder–Davis–Gundy inequality ([11, Theorem 10.36]) giving $c_2> 0$ and the first line of the following inequality. Then, we pull out $\sup _{s\in {[t,T]}} |Y_s|^{\frac{p}{2}}$ from the *ds*-integral (and the squareroot) and finally use Young’s inequality for arbitrary $R > 0$, to estimate

19 $$\begin{aligned}&{\mathbb {E}}\sup _{r\in [t,T]}\left| \int _{t}^{r} e^{\int _0^s\eta (\tau )d\tau }p {\langle Y_{s}|Y_{s}|^{p-2} , Z_s dW_s \rangle } \right| \nonumber \\&\quad \le c_2{\mathbb {E}}\left( \int _t^T (e^{\int _0^s\eta (\tau )d\tau }p |Y_{s}|^{p-1} |Z_s|)^2 ds\right) ^{1/2}\nonumber \\&\quad \le c_2p {\mathbb {E}}\sup _{s\in {[t,T]}} |Y_s|^{\frac{p}{2}} \left( \int _t^T e^{2\int _0^s\eta (\tau )d\tau } |Y_s|^{p-2} |Z_s|^2 ds\right) ^{1/2}\nonumber \\&\quad \le c_3{\mathbb {E}}\left( \frac{1}{R} \sup _{s\in {[t,T]}} |Y_s|^{p} + R \int _t^T |Y_s|^{p-2}|Z_s|^2 ds \right) , \end{aligned}$$

for another constant $c_3> 0$.

For the second suprema, we can use [17, Theorem 3.2] to get $c_4> 0$ such that

$$\begin{aligned}&{\mathbb {E}}\sup _{r \in [t,T]} \left| \int _{{]t, r]}\times {\mathbb {R}}_0^d} e^{\int _0^s\eta (\tau )d\tau }{\langle U(s,x) , p Y_{s-}|Y_{s-}|^{p-2} \rangle }{\tilde{N}}(ds,dx)\right| \\&\quad \le c_4{\mathbb {E}}\left( \int _t^T \int _{{\mathbb {R}}_0^d} e^{2\int _0^s\eta (\tau )d\tau } \left( |U(s,x)|p |Y_{s}||Y_{s}|^{p-2}\right) ^2 \nu (dx)ds\right) ^\frac{1}{2} \\&\quad \le c_5{\mathbb {E}}\left( \frac{1}{R} \sup _{s\in {[t,T]}} |Y_s|^{p} + R \int _t^T |Y_s|^{p-2}\Vert U_s\Vert ^2 ds \right) . \end{aligned}$$

In the last step, we used Young’s inequality as above in (19) for some arbitrary $R > 0$ to get the constant $c_5> 0$.

**Step 4:**

With the last step’s results, we continue from (18) to get a constant $D > 0$ satisfying

20 $$\begin{aligned}&{\mathbb {E}}\sup _{s\in {[t,T]}}e^{\int _0^s\eta (\tau )d\tau }{|Y_s|}^p\nonumber \\&\quad \le D {\mathbb {E}}\Biggl ( |\xi |^p+\int _t^T \alpha (s)\rho (|Y_s|^p)ds + I_{|f_0|}^{p} \nonumber \\&\quad \quad +\frac{1}{R} \sup _{s\in {[t,T]}} |Y_s|^{p} + R \int _t^T |Y_s|^{p-2}|Z_s|^2 ds + R\int _t^T |Y_s|^{p-2}\Vert U_s\Vert ^2 ds \Biggr ). \end{aligned}$$

We apply inequality (16) yielding

$$\begin{aligned} {\mathbb {E}}\sup _{s\in {[t,T]}}e^{\int _0^s\eta (\tau )d\tau }{|Y_s|}^p&\le D(1+Rk) {\mathbb {E}}\Biggl ( |\xi |^p+\int _t^T \alpha (s)\rho (|Y_s|^p)ds + I_{|f_0|}^{p}\Biggr ) \\&\quad + \frac{D}{R} {\mathbb {E}}\sup _{s\in {[t,T]}} |Y_s|^{p}. \end{aligned}$$

We choose $R = 2D$, which implies that there is $D_1>0$ such that

$$\begin{aligned} {\mathbb {E}}\sup _{s\in {[t,T]}}{|Y_s|}^p&\le {\mathbb {E}}\sup _{s\in {[t,T]}}e^{\int _0^s\eta (\tau )d\tau }{|Y_s|}^p \\&\le D_1 \Biggl ( {\mathbb {E}}|\xi |^p+ {\mathbb {E}}I_{|f_0|}^{p}+\int _t^T \alpha (s)\rho ({\mathbb {E}}\sup _{r\in {[s,T]}}|Y_r|^p)ds \Biggr ), \end{aligned}$$

where we also used the concavity of $\rho$. Now, the Bihari–LaSalle inequality (see Theorem 5 in Appendix) finishes the proof. $\square$

### Proposition 4

Let $1<p<2$ and let (*Y*, *Z*, *U*) be an $L^p$-solution to the BSDE $(\xi ,f)$. If $\xi \in L^p$ and (A 1) and (${\text {a3}}_{< 2}$) are satisfied, then there exists a function $h:[0,\infty [\rightarrow [0,\infty [$ with $h(x)\rightarrow 0$ as $x\rightarrow 0$ such that

$$\begin{aligned}&\Vert Y\Vert ^p_{{{\mathscr {S}}}^p} +\left\| Z\right\| _{L^p(W) }^p + \left\| U\right\| _{L^p({{\tilde{N}}}) }^p \le h\left( {\mathbb {E}}|\xi |^p+{\mathbb {E}}I_{|f_0|}^p\right) , \end{aligned}$$

where *h* depends on $p, T, \rho , \alpha , \beta _1, \beta _2, \mu$.

### Proof

**Step 1:**

We begin this proof similarly to the case $p\ge 2$: Let $\eta$ be a progressively measurable process in $L^\infty ({\varOmega }; L^1([0,T]))$, which we will determine later. As carried out in detail in [13, Proposition 3], Itô’s formula, applied to the smooth function $u_\varepsilon : x\mapsto (|x|^2+\varepsilon )^\frac{p}{2}$ and taking the limit $\varepsilon \rightarrow 0$ implies that for $c_0= \frac{p(p-1)}{2}$ and $t \in [0,T]$,

$$\begin{aligned}&e^{\int _0^t\eta (\tau )d\tau }|Y_t|^p + \int _t^T e^{\int _0^s\eta (\tau )d\tau }\Big [\eta (s)|Y_s|^p + c_0|Y_s|^{p-2} |Z_s|^2 \chi _{\{ Y_s \ne 0\}} \Big ] ds + P(t) \\&\quad \le M(t) + e^{\int _0^T\eta (\tau )d\tau }|\xi |^p + \int _t^T e^{\int _0^s\eta (\tau )d\tau }p {\langle Y_s |Y_s|^{p-2} , f(s,Y_s,Z_s,U_s) \rangle } ds, \end{aligned}$$

where

$$\begin{aligned} P(t) =&\int _t^T \int _{{\mathbb {R}}_0^d} e^{\int _0^s\eta (\tau )d\tau }\\&\left[ |Y_{s-}+U_s(x)|^p - |Y_{s-}|^p - {\langle U_s(x) , pY_{s-}|Y_{s-}|^{p-2} \rangle }\right] \nu (dx)ds \end{aligned}$$

and

$$\begin{aligned} M(t) =&- \int _t^T e^{\int _0^s\eta (\tau )d\tau }p {\langle Y_{s}|Y_{s}|^{p-2} , Z_s dW_s \rangle } \\&- \int _{{]t, T]}\times {\mathbb {R}}_0^d} e^{\int _0^s\eta (\tau )d\tau }\left[ |Y_{s-}+U_s(x)|^p - |Y_{s-}|^p\right] {\tilde{N}}(ds,dx). \end{aligned}$$

The terms slightly differ from [13, Proposition 3]. The alternative expressions are due to the relation $d\tilde{N}(ds,dx)=dN(ds,dx)-\nu (dx)dt$, used to split up the integrals w.r.t. those random measures accordingly in the limit procedure. We may do this as all relevant integrands appearing in the Itô formula for $u_\varepsilon$ and in the limit expression yield $\mathbb {P}$-a.s. finite integrals. Their finiteness results from the convexity of $u_\varepsilon$, the boundedness of its second derivative for all $\varepsilon >0$ and from the fact that $|Y_{s-}|\vee |Y_{s-}+U_s(x)|\le 4\sup _{t\in [0,T]}|Y_t|$.

By the argument in [13, Proposition 3] we can use the estimate

$$\begin{aligned} P(t)&\ge c_0\int _t^T e^{\int _0^s\eta (\tau )d\tau }\\&\quad \int _{{\mathbb {R}}_0^d}(|Y_{s-}|\vee |Y_{s-}+U_s(x)|)^{p-2} |U_s(x)|^2\chi _{(|Y_{s-}|\vee |Y_{s-}+U_s(x)|) \ne 0\}}\nu (dx) ds \, \end{aligned}$$

leading to

$$\begin{aligned}&e^{\int _0^t\eta (\tau )d\tau }|Y_t|^p + \int _t^T e^{\int _0^s\eta (\tau )d\tau }\Big [\eta (s)|Y_s|^p + c_0|Y_s|^{p-2} |Z_s|^2\chi _{\{ Y_s \ne 0\}} \Big ] ds \\&\qquad +c_0\int _t^T e^{\int _0^s\eta (\tau )d\tau }\\&\qquad \int _{{\mathbb {R}}_0^d}(|Y_{s-}|\vee |Y_{s-}+U_s(x)|)^{p-2} |U_s(x)|^2\chi _{(|Y_{s-}|\vee |Y_{s-}+U_s(x)|) \ne 0\}}\nu (dx)ds \\&\quad \le M(t) + e^{\int _0^T\eta (\tau )d\tau }|\xi |^p + \int _t^T e^{\int _0^s\eta (\tau )d\tau }p {\langle Y_s |Y_s|^{p-2} , f(s,Y_s,Z_s,U_s) \rangle } ds. \end{aligned}$$

Using (${\text {a3}}_{< 2}$) and Young’s inequality, we obtain for an arbitrary $R_z > 0$,

21 $$\begin{aligned}&e^{\int _0^t\eta (s)ds}{|Y_t|}^p+\int _t^T e^{\int _0^s\eta (\tau )d\tau }\left( \eta (s){|Y_s|}^p +c_0|Y_s|^{p-2}|Z_s|^2\chi _{\{ Y_s \ne 0\}}\right) ds\nonumber \\&\qquad +c_0\int _t^T e^{\int _0^s\eta (\tau )d\tau }\nonumber \\&\qquad \int _{{\mathbb {R}}_0^d}(|Y_{s-}|\vee |Y_{s-}+U_s(x)|)^{p-2} |U_s(x)|^2\chi _{(|Y_{s-}|\vee |Y_{s-}+U_s(x)|) \ne 0\}}\nu (dx)ds\nonumber \\&\quad \le e^{\int _0^T\eta (s)ds}|\xi |^p+\int _t^T e^{\int _0^s\eta (\tau )d\tau }p \nonumber \\&\qquad \biggl ( \alpha (s)\rho (|Y_s|^p) +\left( \mu (s)+\frac{R_z \beta _1(s)^2}{2}+(p-1)K_{|f_0|}(s)\right) |Y_s|^p \biggr )ds \nonumber \\&\qquad + \int _t^T e^{\int _0^s\eta (\tau )d\tau }\frac{p}{2 R_z}|Y_s|^{p-2}|Z_s| \chi _{\{ Y_s \ne 0\}}ds \nonumber \\&\qquad +\int _t^T e^{\int _0^s\eta (\tau )d\tau }p\beta _2(s)|Y_s|^{p-1}\Vert U_s\Vert ds\nonumber \\&\qquad + \int _t^T e^{\int _0^s\eta (\tau )d\tau } |f_0(s)| I_{|f_0|}^{p-1}ds+M(t). \end{aligned}$$

We choose $R_z=\frac{p}{c_0}$ and $\eta =p\left( \mu +\frac{R_z \beta _1^2}{2}+(p-1)K_{|f_0|}\right)$, take expectations and omit the first term to arrive at

$$\begin{aligned}&\frac{c_0}{2}{\mathbb {E}}\int _t^T e^{\int _0^s\eta (\tau )d\tau }|Y_s|^{p-2}|Z_s|^2\chi _{\{ Y_s \ne 0\}}ds \\&\qquad +c_0{\mathbb {E}}\int _t^T e^{\int _0^s\eta (\tau )d\tau }\\&\qquad \int _{{\mathbb {R}}_0^d}(|Y_{s-}|\vee |Y_{s-}+U_s(x)|)^{p-2} |U_s(x)|^2 \chi _{(|Y_{s-}|\vee |Y_{s-}+U_s(x)|) \ne 0\}}\nu (dx)ds\\&\quad \le {\mathbb {E}}e^{\int _0^T\eta (s)ds}|\xi |^p+{\mathbb {E}}\int _t^T e^{\int _0^s\eta (\tau )d\tau }p \alpha (s)\rho (|Y_s|^p) ds \\&\qquad +{\mathbb {E}}\int _t^T e^{\int _0^s\eta (\tau )d\tau }p\beta _2(s)|Y_s|^{p-1}\Vert U_s\Vert ds+ {\mathbb {E}}\int _t^T e^{\int _0^s\eta (\tau )d\tau } |f_0(s)| I_{|f_0|}^{p-1}ds, \end{aligned}$$

yielding a constant $C>0$ such that

22 $$\begin{aligned}&{\mathbb {E}}\int _t^T |Y_s|^{p-2}|Z_s|^2\chi _{\{ Y_s \ne 0\}}ds\nonumber \\&\qquad +{\mathbb {E}}\int _t^T \int _{{\mathbb {R}}_0^d}(|Y_{s-}|\vee |Y_{s-}+U_s(x)|)^{p-2} |U_s(x)|^2 \chi _{(|Y_{s-}|\vee |Y_{s-}+U_s(x)|) \ne 0\}}\nu (dx)ds\nonumber \\&\quad \le C\biggl ({\mathbb {E}}|\xi |^p+{\mathbb {E}}\int _t^T \alpha (s)\rho (|Y_s|^p) ds +{\mathbb {E}}\int _t^T \beta _2(s)|Y_s|^{p-1}\Vert U_s\Vert ds+ {\mathbb {E}}I_{|f_0|}^p\biggr ). \end{aligned}$$

**Step 2:**

In this step, we leave the argumentation lines of Kruse and Popier [13, 14] and Briand et al [3], estimating several terms differently and using the integrability assumptions on $\beta _2$. We start from estimating the suprema of the stochastic integrals appearing in (21) by similar means as in step 3 of the proof of Proposition 3, (18) - (20) which yields constants $c,c_1> 0$, such that for an arbitrary $R>0$ we get

23 $$\begin{aligned}&\sup _{s\in [t,T]}|M(s)| \nonumber \\&\quad \le c\biggl (\frac{1}{R}{\mathbb {E}}\sup _{s\in {[t,T]}}|Y_s|^p+R{\mathbb {E}}\int _t^T |Y_s|^{p-2}|Z_s|^2\chi _{\{Y_s\ne 0\}}ds\nonumber \\&\qquad +{\mathbb {E}}\biggl [\int _t^T\int _{{\mathbb {R}}_0^d}\left( \left( |Y_{s-}+U_s(x)|\vee |Y_{s-}|\right) ^{p-1}|U_s(x)|\right) ^2\chi _{(|Y_{s-}|\vee |Y_{s-}+U_s(x)|) \ne 0\}}\nu (dx)ds\biggr ]^\frac{1}{2}\biggr )\nonumber \\&\quad \le c_1\biggl (\frac{1}{R}{\mathbb {E}}\sup _{s\in {[t,T]}}|Y_s|^p+R{\mathbb {E}}\int _t^T |Y_s|^{p-2}|Z_s|^2\chi _{\{Y_s\ne 0\}}ds\nonumber \\&\qquad +R{\mathbb {E}}\int _t^T\int _{{\mathbb {R}}_0^d}\left( |Y_{s-}+U_s(x)|\vee |Y_{s-}|\right) ^{p-2}|U_s(x)|^2\chi _{(|Y_{s-}|\vee |Y_{s-}+U_s(x)|) \ne 0\}}\nu (dx)ds\biggr ), \end{aligned}$$

where again we used Young’s inequality as well as that $|Y_{s-}+U_s(x)|\vee |Y_{s-}|\le 4\sup _{s\in {[t,T]}}|Y(s)|$, $\mathbb {P}\otimes \lambda \otimes \nu$-a.e.

Taking suprema in (21) with the same choices $R_z=\frac{p}{c_0}$ and $\eta =p\left( \mu +\frac{R_z \beta _1^2}{2}+(p-1)K_{|f_0|}\right)$ (to cancel out appropriate terms) yields

$$\begin{aligned}&\sup _{s\in {[t,T]}}e^{\int _0^s\eta (\tau )d\tau }{|Y_s|}^p \\&\qquad +c_0\int _t^T e^{\int _0^s\eta (\tau )d\tau }\\&\qquad \int _{{\mathbb {R}}_0^d}(|Y_{s-}|\vee |Y_{s-}+U_s(x)|)^{p-2} |U_s(x)|^2\chi _{(|Y_{s-}|\vee |Y_{s-}+U_s(x)|) \ne 0\}}\nu (dx)ds\\&\quad \le e^{\int _0^T\eta (s)ds}|\xi |^p+\int _t^T e^{\int _0^s\eta (\tau )d\tau }p \alpha (s)\rho (|Y_s|^p)ds \\&\qquad +\int _t^T e^{\int _0^s\eta (\tau )d\tau }p\beta _2(s)|Y_s|^{p-1}\Vert U_s\Vert ds\\&\qquad + \int _t^T e^{\int _0^s\eta (\tau )d\tau } |f_0(s)| I_{|f_0|}^{p-1}ds+\sup _{s\in [t,T]}|M(s)|, \end{aligned}$$

where we omitted the remaining positive integral terms $\frac{c_0}{2}{\mathbb {E}}\int _t^T e^{\int _0^s\eta (\tau )d\tau } |Y_s|^{p-2}|Z_s|^2\chi _{\{ Y_s \ne 0\}}ds$ and

$$\begin{aligned} c_0\int _t^T e^{\int _0^s\eta (\tau )d\tau }\int _{{\mathbb {R}}_0^d}(|Y_{s-}|\vee |Y_{s-}+U_s(x)|)^{p-2} |U_s(x)|^2\chi _{(|Y_{s-}|\vee |Y_{s-}+U_s(x)|) \ne 0\}}\nu (dx)ds \end{aligned}$$

that appear on the left-hand side after cancelling the integrals involving $|Y_s|^{p-2}|Z_s|^2$ and $|Y(s)|^p$ (not the one with $\rho$) on the right-hand side of (21) after substituting $R_z$ and $\eta$.

Using the above estimate (23) for $\sup _{s\in [t,T]}|M(s)|$, we take expectations and come to

$$\begin{aligned}&{\mathbb {E}}\sup _{s\in {[t,T]}}e^{\int _0^s\eta (\tau )d\tau }{|Y_s|}^p \\&\quad \le {\mathbb {E}}e^{\int _0^T\eta (s)ds}|\xi |^p+{\mathbb {E}}\int _t^T e^{\int _0^s\eta (\tau )d\tau }p \alpha (s)\rho (|Y_s|^p)ds \\&\qquad +{\mathbb {E}}\int _t^T e^{\int _0^s\eta (\tau )d\tau }p|Y_s|^{p-1}\beta _2(s)\Vert U_s\Vert ds+ {\mathbb {E}}\int _t^T e^{\int _0^s\eta (\tau )d\tau } |f_0(s)| I_{|f_0|}^{p-1}ds\\&\qquad +\frac{c_1}{R}\sup _{s\in {[t,T]}}{|Y_s|}^p+c_1R\\&\qquad \biggl ({\mathbb {E}}\int _t^T |Y_s|^{p-2}|Z_s|^2\chi _{\{ Y_s \ne 0\}}ds +{\mathbb {E}}\int _t^T \\&\quad \qquad \int _{{\mathbb {R}}_0^d}(|Y_{s-}|\vee |Y_{s-}+U_s(x)|)^{p-2} |U_s(x)|^2 \chi _{(|Y_{s-}|\vee |Y_{s-}+U_s(x)|) \ne 0\}}\nu (dx)ds\biggr ). \end{aligned}$$

Now inequality (22) can be plugged in for the last parentheses to estimate, for another constant $D>0$,

24 $$\begin{aligned}&{\mathbb {E}}\sup _{s\in {[t,T]}}{|Y_s|}^p \nonumber \\&\quad \le D\biggl ((1+R){\mathbb {E}}|\xi |^p+(1+R){\mathbb {E}}\int _t^T \alpha (s)\rho (|Y_s|^p)ds \nonumber \\&\qquad \quad +(1+R){\mathbb {E}}\int _t^T\beta _2(s)|Y_s|^{p-1}\Vert U_s\Vert ds \nonumber \\&\qquad \quad + (1+R){\mathbb {E}}\int _t^T |f_0(s)| I_{|f_0|}^{p-1}ds+\frac{1}{R}{\mathbb {E}}\sup _{s\in {[t,T]}}{|Y_s|}^p\biggr ). \end{aligned}$$

We focus on the term ${\mathbb {E}}\int _t^T\beta _2(s)|Y_s|^{p-1}\Vert U_s\Vert ds$, which we estimate by

$$\begin{aligned}&{\mathbb {E}}\sup _{s\in {[t,T]}}|Y_s|^{p-1}\int _t^T\beta _2(s)\Vert U_s\Vert ds\\&\quad \le \frac{c_2}{R_1}{\mathbb {E}}\sup _{s\in {[t,T]}}|Y_s|^p+ c_2R_1^{p-1}{\mathbb {E}}\left[ \int _t^T\beta _2(s)\Vert U_s\Vert ds\right] ^p, \end{aligned}$$

for $R_1 > 0$ and a constant $c_2>0$ coming from Young’s inequality for the couple $(p,\frac{p}{p-1})$. By the Cauchy–Schwarz inequality, we get

25 $$\begin{aligned}&{\mathbb {E}}\sup _{s\in {[t,T]}}|Y_s|^{p-1}\int _t^T\beta _2(s)\Vert U_s\Vert ds\nonumber \\&\quad \le \frac{c_2}{R_1}{\mathbb {E}}\sup _{s\in {[t,T]}}|Y_s|^p+2 c_2R_1^{p-1}{\mathbb {E}}\left[ \left( \int _t^T\beta _2(s)^2ds \right) ^\frac{p}{2}\left( \int _t^T\Vert U_s\Vert ^2 ds\right) ^\frac{p}{2}\right] . \end{aligned}$$

Now we use the additional integrability of $\beta _2$ with a power $q>2$. For the case $\beta _2 \in L^2([0,T])$, see Remark 5 after the proof. Here, in the sequel we treat a nondeterministic $\beta _2$ where higher integrability is needed. It has the only purpose to obtain a factor containing $T-t$ by Hölder’s inequality. Indeed, we infer from (25)

$$\begin{aligned}&{\mathbb {E}}\sup _{s\in {[t,T]}}|Y_s|^{p-1}\int _t^T\beta _2(s)\Vert U_s\Vert ds\\&\quad \le \frac{c_2}{R_1}{\mathbb {E}}\sup _{s\in {[t,T]}}|Y_s|^p+2 c_2R_1^{p-1} \\&\qquad {\mathbb {E}}\left[ (T-t)^{\frac{pq}{q-2}}\left( \int _t^T\beta _2(s)^qds \right) ^\frac{p}{q}\left( \int _t^T\Vert U_s\Vert ^2 ds\right) ^\frac{p}{2}\right] . \end{aligned}$$

Now, by the boundedness of $\int _0^T\beta _2(s)^qds$, and applying Proposition 2, we get a constant $D_1>0$ such that

26 $$\begin{aligned}&{\mathbb {E}}\sup _{s\in {[t,T]}}|Y_s|^{p-1}\int _t^T\beta _2(s)\Vert U_s\Vert ds \nonumber \\&\quad \le \frac{c_2}{R_1}{\mathbb {E}}\sup _{s\in {[t,T]}}|Y_s|^p+D_1 R_1^{p-1}(T-t)^{\frac{pq}{q-2}} \nonumber \\&\qquad \left( {\mathbb {E}}\sup _{s\in {[t,T]}}|Y_s|^{p}+{\mathbb {E}}\int _t^T\alpha (s)\rho (|Y(s)|^p)ds+{\mathbb {E}}I_{|f_0|}^p\right) . \end{aligned}$$

Inserting (26) into inequality (24), we get for a new constant ${\tilde{D}} > 0$

$$\begin{aligned} {\mathbb {E}}\sup _{s\in {[t,T]}}{|Y_s|}^p&\le {\tilde{D}} \Biggl ((1+R) {\mathbb {E}}|\xi |^p+(1+R){\mathbb {E}}\int _t^T \alpha (s)\rho (|Y_s|^p)ds + (1+R){\mathbb {E}}I_{|f_0|}^p \\&\quad +\left( \frac{1}{R}+\frac{(1+R)c_2}{R_1}+(1+R)D_1R_1^{p-1}(T-t)^\frac{pq}{q-2}\right) {\mathbb {E}}\sup _{s\in {[t,T]}}{|Y_s|}^p\Biggr ). \end{aligned}$$

Now, choose *R* such that $\frac{{\tilde{D}}}{R}<\frac{1}{2}$, afterwards choose $R_1$ such that $\frac{{\tilde{D}}(1+R)c_2}{R_1}<\frac{1}{4}$. Now, our goal for the next step is to divide [0, *T*] into small parts in order to make the third term containing $(T-t)$ small too.

**Step 3:**

From here on, let the time interval [0, *T*] be partitioned into $0=t_0<t_1<\dotsc <t_n=T$, such that for all $1\le i\le n$, ${\tilde{D}}(1+R)D_1R_1^{p-1}(t_i-t_{i-1})^\frac{pq}{q-2}<\frac{1}{8}$. Thus, on the interval $[t_{n-1},T]$, we come to a constant $D_2>0$ such that

$$\begin{aligned} {\mathbb {E}}\sup _{s\in {[t,T]}}{|Y_s|}^p \le D_2\biggl ({\mathbb {E}}|\xi |^p+\int _t^T \alpha (s)\rho ({\mathbb {E}}|Y_s|^p)ds +{\mathbb {E}}I_{|f_0|}^p\biggr ). \end{aligned}$$

Now, the Bihari–LaSalle inequality (Theorem 5) shows that there is a function $h_n$ such that

$$\begin{aligned} {\mathbb {E}}\sup _{s\in {[t_{n-1},T]}}{|Y_s|}^p\le h_n({\mathbb {E}}|\xi |^p+{\mathbb {E}}I_{|f_0|}^p). \end{aligned}$$

Performing the same steps as above for the interval $[t_{n-2},t_{n-1}]$, we find a function $h_{n-1}$ such that

$$\begin{aligned} {\mathbb {E}}\sup _{s\in {[t_{n-2},t_{n-1}]}}{|Y_s|}^p\le h_{n-1}({\mathbb {E}}|Y_{t_{n-1}}|^p+{\mathbb {E}}I_{|f_0|}^p)\le h_{n-1}\bigl (h_n({\mathbb {E}}|\xi |^p+{\mathbb {E}}I_{|f_0|}^p)+{\mathbb {E}}I_{|f_0|}^p\bigr ). \end{aligned}$$

Iterating the procedure backwards in time, we end up with functions $h_1,\dotsc ,h_n$, accumulating to a function ${\tilde{h}}$, such that

$$\begin{aligned} {\mathbb {E}}\sup _{s\in {[0,T]}}{|Y_s|}^p\le {\tilde{h}}({\mathbb {E}}|\xi |^p+{\mathbb {E}}I_{|f_0|}^p). \end{aligned}$$

The bound for $\Vert Z\Vert ^p_{L^p(W)}+ \Vert U\Vert ^p_{L^p({\tilde{N}})}$ then follows from Proposition 2, concluding the proof. $\square$

### Remark 5

In step 3, if $\beta _2$ is deterministic, we could impose the weaker condition, namely $\beta _2$ being only square-integrable (instead of in $L^q$, for some $q > 2$). Then we do not need to apply Hölder’s inequality to (25) in order to choose the division of [0, *T*] such that $D_1(t_i-t_{i-1})^\frac{pq}{q-2}$ is small. Instead we choose the partition such that the $\int _{t_{i-1}}^{t_i}\beta _2(s)^2ds$ become sufficiently small.

With the technique from the two above a priori estimates in hand, we can now prove another key part for the existence proof: boundedness stability of the *Y* process, meaning that the solution process *Y* stays bounded, when the data ($\xi$, *f*) have boundedness properties:

### Proposition 5

Let $$p>1 $$ and (*Y*, *Z*, *U*) be an $$L^p$$-solution to the BSDE $$(\xi ,f)$$.

If $$\xi ,I_{|f_0|}\in L^\infty $$ and

(i) for $p\ge 2$, (A 1) and (${\text {a3}}_{\ge 2}$) hold,
(ii) or for $1< p < 2$, (A 1) and (${\text {a3}}_{< 2}$) hold,

then there exists a constant $C > 0$ such that for all $t\in {[0,T]}$,

$$\begin{aligned}|Y_t|^p\le C,\quad {\mathbb {P}}\text {-a.s.}\end{aligned}$$

Here, *C* depends on $\Vert \xi \Vert _\infty ,\Vert I_{|f_0|}\Vert _\infty ,\mu ,p,T,\rho ,\alpha ,\beta$ for $p \ge 2$. If $p<2$, C depends on the same variables but $\beta _1,\beta _2,q$ instead of $\beta$.

### Proof

We copy the proofs of Proposition 3 and Proposition 4 for the cases $1<p<2$ and $2\le p$, replacing the operator ${\mathbb {E}}$ by ${\mathbb {E}}\left[ \ \cdot \ \big | {{\mathscr {F}}}_t\right]$ considering the BSDEs on [*t*, *T*], which leads to the estimates ${\mathbb {E}}\left[ \sup _{s\in {[t,T]}}|Y_s|^p \big | {{\mathscr {F}}}_t\right] <C$ for all $t\in [0,T]$. The assertion now follows from the monotonicity of the conditional expectation. $\square$

## Proof of the Main Theorem 1

The proof basically follows the one in Briand et al. [3, Theorem 4.2]. For convenience of the reader, we give a detailed proof adapted to our more general setting. We consider only the case $1<p< 2$ as the case $p \ge 2$ is similar but easier.

**Step 1:** Uniqueness

Assume we have another solution $(Y',Z',U')$. Then Proposition 4 applied to the BSDE (0, *g*) with $g(t,y,z,u)=f(t,y+Y',z+Z',u+U')-f(Y',Z',U')$ implies $(Y-Y',Z-Z',U-U')=(0,0,0)$.

**Step 2:**

In this step, we construct a first approximating sequence of generators for *f* and show several estimates for the solution processes. Assume that $\xi , I_{|f_0|}\in L^\infty$. As (${\text {A3}}_{< 2}$) is satisfied, the condition is also satisfied for the changed parameter $\mu ' = \rho (1)\alpha +\mu$. We take the constant *C* appearing in Proposition 5 and choose an $r>C$.

Take a smooth real function $\theta _r$ such that $0\le \theta _r\le 1$, $\theta _r(y)=1$ for $|y|\le r$ and $\theta _r(y)=0$ for $|y|\ge r+1$ and define

$$\begin{aligned} h_n(t,y,z,u):=\theta _r(y)\left( f(t,y,c_n(z),{\tilde{c}}_n(u))-f_0(t)\right) \frac{n}{\psi _{r+1}(t)\vee n}+f_0(t). \end{aligned}$$

Here, $c_n, {\tilde{c}}_n$ are the projections $x \mapsto nx/(|x| \vee n)$ onto the closed unit balls of radius *n*, respectively, in ${\mathbb {R}}^{d \times k}$ and $L^2(\nu )$.

These generators $h_n$ satisfy the following properties for all $n \in {\mathbb {N}}$:

(A i) Condition (A 1) is satisfied.
(A ii) By (A 2),
  $$\begin{aligned} |h_n(t,y,z,u)|\le n + |f_0(t)| + {\varPhi }(t)(|z|+\Vert u\Vert ). \end{aligned}$$
(A iii) By (A 2) and (${\text {A3}}_{< 2}$), with $\beta = \beta _1 + \beta _2$, and $C_r$ denoting the Lipschitz constant of $\theta _r$, it holds that
  $$\begin{aligned}&{\langle y-y' , h_n(t,y,z,u)-h_n(t,y',z',u') \rangle }\\&\quad =\theta _r(y)\frac{n}{\psi _{r+1}(t)\vee n}{\langle y-y' , f(t,y,c_n(z),{\tilde{c}}_n(u))-f(t,y',c_n(z'),{\tilde{c}}_n(u')) \rangle }\\&\qquad +\frac{n}{\psi _{r+1}(t)\vee n}\left( \theta _r(y)-\theta _r(y')\right) {\langle y-y' , f(t,y',c_n(z'),{\tilde{c}}_n(u'))-f_0(t) \rangle }\\&\quad \le \frac{n}{\psi _{r+1}(t)\vee n} \\&\qquad \left( \alpha (t)\frac{\rho (|y-y'|^p)}{|y-y'|^{p-2}}+\mu (s)|y-y'|^2+\beta (t)(|z-z'|+\Vert u-u'\Vert )\right) \\&\qquad +C_r(2{\varPhi }(s)+1)n|y-y'|^2\\&\quad \le \alpha (t)\rho (|y-y'|^2)+\left( \rho (1)\alpha (s)+\mu (s)+C_r(2{\varPhi }(s)+1)n\right) |y-y'|^2\\&\qquad +\beta (t)(|z-z'|+\Vert u-u'\Vert ), \end{aligned}$$
  where we used Remark 2(ii)(b).
(A iv) By (${\text {A3}}_{< 2}$), again with $\beta = \beta _1 + \beta _2$ we have,
  $$\begin{aligned} yh_n(t,y,z,u)\le |y||f_0(t)|+\alpha (t)\rho (|y|^2)+(\rho (1)\alpha (t)+\mu (t))|y|^2+\beta (t)|y|(|z|+\Vert u\Vert ). \end{aligned}$$

Properties (A i)-(A iii) imply that for $\mu _r(s):=\left( \rho (1)\alpha (s)+\mu (s)+C_r(2{\varPhi }(s)+1)n\right)$ the generator $g_n(t,y,z,u):=e^{\int _0^\cdot \mu _r(s)ds}\left( h_n-\mu _r\cdot y\right)$ satisfies assumptions (A 1)-(A 3) of Theorem 3.1 in [10] (or rather a straightforward adaptation to *d* dimensions of it) and admits a unique solution of BSDE $(e^{\int _0^T \mu _r(s)ds}\xi ,g_n)$. Thus, by the transformation $({\tilde{Y}},{\tilde{Z}},{\tilde{U}}):=e^{-\int _0^\cdot \mu _r(s)ds}(Y,Z,U)$, one gets that also $(\xi ,h_n)$ has a unique solution $(Y^n,Z^n,U^n)$.

Moreover, by property (A iv) and (7) we are able to apply Proposition 5 to get that $\Vert Y_t^n\Vert _\infty \le r$. Since $Y^n_t$ is bounded by *r*, we get that $(Y^n,Z^n,U^n)$ is also a solution to the BSDE $(\xi ,f_n)$, with

$$\begin{aligned} f_n(t,y,z,u):=\left( f(t,y,c_n(z),{\tilde{c}}_n(u))-f_0(t)\right) \frac{n}{\psi _{r+1}(t)\vee n}+f_0(t). \end{aligned}$$

Comparing the solutions $(Y^n,Z^n,U^n)$ and $(Y^m,Z^m,U^m)$ for $m\ge n$, we use the standard methods from (14)-(15), for the differences

$$\begin{aligned} ({\varDelta }Y, {\varDelta }Z, {\varDelta }U):=(Y^m,Z^m,U^m)-(Y^n,Z^n,U^n). \end{aligned}$$

In this procedure, we replace the use of the monotonicity condition (${\text {A3}}_{\ge 2}$) in Proposition 4 by

$$\begin{aligned}&|{\varDelta }Y_s|^{p-2} {\langle {\varDelta }Y_s , f_m(s,Y^m_s,Z^m_s,U^m_s)-f_n(s, Y^n_s,Z^n_s,U^n_s) \rangle } \\&\quad = |{\varDelta }Y_s|^{p-2} {\langle {\varDelta }Y_s , f_m(s,Y^m_s,Z^m_s,U^m_s)-f_m(s, Y^n_s,Z^n_s,U^n_s) \rangle } \\&\qquad + |{\varDelta }Y_s|^{p-2} {\langle {\varDelta }Y_s , f_m(s,Y^n_s,Z^n_s,U^n_s)-f_n(s, Y^n_s,Z^n_s,U^n_s) \rangle } \\&\quad \le \alpha (s)\rho (|{\varDelta }Y_s|^p)+\left( \rho (1)\alpha (s)+\mu (s)+ \frac{R_z+R_u}{2}\beta (s)^2\right) |{\varDelta }Y_s|^p \\&\qquad +\left( \frac{|{\varDelta }Z_s|^2}{2R_z}+\frac{\Vert {\varDelta }U_s\Vert ^2}{2R_u}\right) \\&\qquad + |{\varDelta }Y_s|^{p-2} \left| {\langle {\varDelta }Y_s , f_m(s,Y^n_s,Z^n_s,U^n_s)-f_n(s, Y^n_s,Z^n_s,U^n_s) \rangle }\right| , \end{aligned}$$

such that the same steps of the proof of Proposition 4 can be conducted to get a function *h* with

$$\begin{aligned}&\Vert {\varDelta }Y\Vert ^p_{{{\mathscr {S}}}^p} +\left\| {\varDelta }Z\right\| _{L^p(W) }^p + \left\| {\varDelta }U\right\| _{L^p({{\tilde{N}}}) }^p \\&\quad \le h\left( {\mathbb {E}}\int _0^T |{\varDelta }Y_s|^{p-2} \left| {\langle {\varDelta }Y_s , f_m(s,Y^n_s,Z^n_s,U^n_s)-f_n(s,Y^n_s,Z^n_s,U^n_s) \rangle }\right| ds\right) \end{aligned}$$

(in the case for (${\text {A3}}_{\ge 2}$), we use the steps from Proposition 3 and Proposition 1). So $\Vert {\varDelta }Y\Vert ^p_{{{\mathscr {S}}}^p} +\left\| {\varDelta }Z\right\| _{L^p(W) }^p + \left\| {\varDelta }U\right\| _{L^p({{\tilde{N}}}) }^p$ tends to zero if

$$\begin{aligned} {\mathbb {E}}\int _0^T |{\varDelta }Y_s|^{p-2} \left| {\langle {\varDelta }Y_s , f_m(s,Y^n_s,Z^n_s,U^n_s)-f_n(s, Y^n_s,Z^n_s,U^n_s) \rangle }\right| ds \end{aligned}$$

does, which we will show next (in the case of (${\text {A3}}_{\ge 2}$), this follows from Proposition 3 with (8)).

Since $|Y^m_t|,|Y^n_t|\le r$, we estimate

27 $$\begin{aligned}&{\mathbb {E}}\int _0^T |{\varDelta }Y_s|^{p-2} \left| {\langle {\varDelta }Y_s , f_m(s,Y^n_s,Z^n_s,U^n_s)-f_n(s, Y^n_s,Z^n_s,U^n_s) \rangle }\right| ds\nonumber \\&\quad \le (2r)^{p-1}{\mathbb {E}}\int _0^T \left| f_m(s,Y^n_s,Z^n_s,U^n_s)-f_n(s, Y^n_s,Z^n_s,U^n_s)\right| ds. \end{aligned}$$

Because of the definition of $f_m, f_n$ and since $m \ge n$, the integrand is zero if $|Z_s|\le n, \Vert U_s\Vert \le n$ and $\psi _{r+1}(s)\le n$ and bounded by

28 $$\begin{aligned}&2{\varPhi }(s)\left( |Z^n_s|+\Vert U^n_s\Vert \right) \chi _{\{|Z^n_s|+\Vert U^n_s\Vert>n\}}+2{\varPhi }(s)\left( |Z^n_s|+\Vert U^n_s\Vert \right) \chi _{\{\psi _{r+1}(s)>n\}}\nonumber \\&\quad +2\psi _{r+1}(s)\chi _{\{\psi _{r+1}(s)>n\}}+2\psi _{r+1}(s)\chi _{\{|Z^n_s|+\Vert U^n_s\Vert >n\}} \end{aligned}$$

otherwise.

To show convergence of the integral of (28), we use the uniform integrability of the families ${\varPhi }(|Z^n|+\Vert U^n\Vert )_{n\ge 1}$ with respect to the measure ${\mathbb {P}}\otimes \lambda$, which follows from

$$\begin{aligned}&{\mathbb {E}}\int _0^T{\varPhi }(s)(|Z^n_s|+\Vert U^n_s\Vert )ds\le {\mathbb {E}}\int _0^T \left( {\varPhi }(s)^2+|Z^n_s|^2+\Vert U^n_s\Vert ^2\right) ds\\&\quad \le \left\| \int _0^T {\varPhi }(s)^2ds\right\| _\infty +r', \end{aligned}$$

since by Proposition 4 and (A iv), there is $r'>0$ such that $\left\| Z^n\right\| _{L^2(W) }^2 + \left\| U^n\right\| _{L^2({{\tilde{N}}}) }^2 < r'$. Therefore, as (28) (as sequence in *n*) is uniformly integrable with respect to ${\mathbb {P}}\otimes \lambda$, dominating the sequence $\left( \left| f_m(s,Y^n_s,Z^n_s,U^n_s)-f_n(s, Y^n_s,Z^n_s,U^n_s)\right| \right) _{n\ge 0}$, which approaches zero pointwisely, also (27) tends to zero as $m>n\rightarrow \infty$. Hence, also $\Vert {\varDelta }Y\Vert ^p_{{{\mathscr {S}}}^p} +\left\| {\varDelta }Z\right\| _{L^p(W) }^p + \left\| {\varDelta }U\right\| _{L^p({{\tilde{N}}}) }^p$ tend to zero, showing that the $(Y^n,Z^n,U^n)$ form a Cauchy sequence in ${{\mathscr {S}}}^p\times L^p(W)\times L^p({\tilde{N}})$ and converge to an element (*Y*, *Z*, *U*).

**Step 3:**

We now show that (*Y*, *Z*, *U*) satisfies the BSDE $(\xi , f)$, for $\xi , I_{|f_0|}\in L^\infty$, as supposed in step 2. The stochastic integral terms of the BSDEs $(\xi ,f_n)$ with solution $(Y^n,Z^n,U^n)$ converge to the corresponding terms of the BSDE $(\xi ,f)$ also in probability. It is left to show that, at least for a subsequence,

$$\begin{aligned} \int _t^T f_n(s,Y^n_s,Z^n_s,U^n_s)ds\rightarrow \int _t^T f(s,Y_s,Z_s,U_s)ds,\quad {\mathbb {P}}\text {-a.s.} \end{aligned}$$

For an appropriate subsequence, all other terms of the BSDEs converge almost surely. W.l.o.g, this subsequence is assumed to be the original one. Hence, we know that there is a random variable $V_t$ such that

$$\begin{aligned} \int _t^T f_n(s,Y^n_s,Z^n_s,U^n_s)ds\rightarrow V_t,\quad {\mathbb {P}}\text {-a.s.} \end{aligned}$$

We take expectations and split up the integral into

$$\begin{aligned} \delta ^{(1)}:={\mathbb {E}}\int _t^T \left( f_n(s,Y^n_s,Z^n_s,U^n_s)-f(s,Y^n_s,Z^n_s,U^n_s)\right) ds \end{aligned}$$

and

$$\begin{aligned} \delta ^{(2)}:={\mathbb {E}}\int _t^T \left( f(s,Y^n_s,Z^n_s,U^n_s)-f(s,Y_s,Z_s,U_s)\right) ds. \end{aligned}$$

By the same argument as for inequality (28),

$$\begin{aligned} |\delta ^{(1)}|&\le {\mathbb {E}}\int _0^T \\&\quad \biggl [2{\varPhi }(s)\left( |Z^n_s|+\Vert U^n_s\Vert \right) \chi _{\{|Z^n_s|+\Vert U^n_s\Vert>n\}}\\&\qquad +2{\varPhi }(s)\left( |Z^n_s|+\Vert U^n_s\Vert \right) \chi _{\{\psi _{r+1}(s)>n\}} \\&\qquad +2\psi _{r+1}(s)\chi _{\{\psi _{r+1}(s)>n\}}+2\psi _{r+1}(s)\chi _{\{|Z^n_s|+\Vert U^n_s\Vert >n\}}\biggr ]ds, \end{aligned}$$

which converges to zero. Now, for $\delta ^{(2)}$, we know that $(Y^n)\rightarrow Y$ in ${{\mathscr {S}}}^p$; hence, there is a subsequence $(Y^{n_\ell })_\ell \ge 1$ s.t. $(Y^{n_\ell }\rightarrow Y)$ uniformly in *t*, ${\mathbb {P}}$-a.s. Since all $Y^{n_l}$ are càdlàg (as *Y*-components of solutions to BSDEs) and bounded by *r* from step 2, we also get that the limit *Y* is càdlàg and $\Vert Y_t\Vert _\infty$ is bounded by *r* for all $t\in {[0,T]}$. Further, we know that $(Y^n,Z^n,U^n)\rightarrow (Y,Z,U)$ in the measure ${\mathbb {P}}\otimes \lambda$. Thus, also $f(s,Y^n_s,Z^n_s,U^n_s)\rightarrow f(s,Y_s,Z_s,U_s)$ in ${\mathbb {P}}\otimes \lambda$. Since now

$$\begin{aligned} \delta ^{(2)}&={\mathbb {E}}\int _t^T \left( f(s,Y^n_s,Z^n_s,U^n_s)-f(s,Y_s,Z_s,U_s)\right) ds\\&={\mathbb {E}}\int _t^T \left( f(s,Y^n_s,Z^n_s,U^n_s)-f_0(s)-(f(s,Y_s,Z_s,U_s)-f_0(s))\right) ds \end{aligned}$$

and, by (A 2),

$$\begin{aligned}&|f(s,Y^n_s,Z^n_s,U^n_s)-f_0(s)|+|f(s,Y_s,Z_s,U_s)-f_0(s)|\\&\quad \le 2\psi _{r+1}(s)+{\varPhi }(s)\left( |Z^n_s|+|Z_s|+\Vert U^n_s\Vert +\Vert U_s\Vert \right) , \end{aligned}$$

from the uniform integrability of $\left( \psi _{r+1}+{\varPhi }(|Z^n|+|Z|+\Vert U^n\Vert +\Vert U\Vert )\right) _{n\ge 1}$ with respect to ${\mathbb {P}}\otimes \lambda$, it follows that also $|\delta ^{(2)}|\rightarrow 0$.

Thus,

$$\begin{aligned} {\mathbb {E}}\int _t^T f_n(s,Y^n_s,Z^n_s,U^n_s)ds\rightarrow {\mathbb {E}}\int _t^T f(s,Y_s,Z_s,U_s)ds, \end{aligned}$$

and extracting a subsequence $(n_l)_{l\ge 1}$ satisfying ${\mathbb {P}}$-a.s

$$\begin{aligned} \int _t^T f_{n_l}(s,Y^{n_l}_s,Z^{n_l}_s,U^{n_l}_s)ds\rightarrow \int _t^T f(s,Y_s,Z_s,U_s)ds, \end{aligned}$$

shows that $V_t=\int _t^T f(s,Y_s,Z_s,U_s)ds$. So (*Y*, *Z*, *U*) satisfies the BSDE $(\xi ,f)$.

**Step 4:**

We now approximate a general $\xi \in L^p$ by $c_n(\xi )$ and the generator *f* by

$$\begin{aligned} f^n(t,y,z,u):=f(t,y,z,u)-f_0(t)+c_n(f_0(t)). \end{aligned}$$

A solution $(Y^n,Z^n,U^n)$ to $(c_n(\xi ),f^n)$ exists due to the last step. Now we get, for $m\ge n$, denoting differences again by

$$\begin{aligned} ({\varDelta }Y, {\varDelta }Z, {\varDelta }U):=(Y^m,Z^m,U^m)-(Y^n,Z^n,U^n) \end{aligned}$$

via Proposition 4 (we use the generator $g_{m,n}(t,y,z,u):=f^m(t,y+Y^n,z+Z^n,u+U^n)-f^n(Y^n,Z^n,U^n)$):

$$\begin{aligned}&\Vert {\varDelta }Y\Vert ^p_{{{\mathscr {S}}}^p} +\left\| {\varDelta }Z\right\| _{L^2(W) }^2 + \left\| {\varDelta }U\right\| _{L^2({{\tilde{N}}}) }^2 \\&\quad \le h\Biggl ({\mathbb {E}}|c_m(\xi )-c_n(\xi )|^p+{\mathbb {E}}\left( \int _0^T \left| f^m(s,Y^n_s,Z^n_s,U^n_s)-f^n(Y^n_s,Z^n_s,U^n_s)\right| ds\right) ^{p}\Biggr )\\&\quad =h\left( {\mathbb {E}}|c_m(\xi )-c_n(\xi )|^p+{\mathbb {E}}\left( \int _0^T \left| c_m(f_0(s))-c_n(f_0(s))\right| ds\right) ^{p}\right) . \end{aligned}$$

As $n\rightarrow \infty$, the latter term tends to zero showing convergence of the sequence $(Y^n,Z^n,U^n)$ to (*Y*, *Z*, *U*) in ${{{\mathscr {S}}}^p}\times {L^p(W) }\times L^p({{\tilde{N}}})$. Again it remains to check that

$$\begin{aligned} \int _t^T f^n(s,Y^n_s,Z^n_s,U^n_s)ds\rightarrow \int _t^T f(s,Y_s,Z_s,U_s)ds, \quad {\mathbb {P}}\text {-a.s.,} \end{aligned}$$

at least for a subsequence. This is equivalent to,

$$\begin{aligned}&\int _t^T \left( f^n(s,Y^n_s,Z^n_s,U^n_s)-f(s,Y^n_s,Z^n_s,U^n_s)\right) ds \\&\quad - \int _t^T \left( f(s,Y_s,Z_s,U_s)-f(s,Y^n_s,Z^n_s,U^n_s)\right) ds \rightarrow 0, \quad {\mathbb {P}}\text {-a.s.,} \end{aligned}$$

The first integral is

$$\begin{aligned} \int _t^T \left( f^n(s,Y^n_s,Z^n_s,U^n_s)-f(s,Y^n_s,Z^n_s,U^n_s)\right) ds =\int _t^T \left( c_n(f_0(s))-f_0(s)\right) ds, \end{aligned}$$

which tends to zero as $n\rightarrow \infty$.

The second integral is more complicated. Here, we extract a subsequence $(n_l)_{l\ge 1}$ such that

$$\begin{aligned}\sup _{t\in [0,T]}|Y_t-Y^{n_l}_t|\rightarrow 0,\quad {\mathbb {P}}\text {-a.s.},\quad (Z^{n_l},U^{n_l})\rightarrow (Z,U),\quad \lambda \text {-a.e.},{\mathbb {P}}\text {-a.s.}\end{aligned}$$

and

$$\begin{aligned} \left( \int _0^T |Z^{n_l}_s|^2 ds,\int _0^T \Vert U^{n_l}_s\Vert ^2 ds\right) \rightarrow \left( \int _0^T |Z_s|^2 ds,\int _0^T \Vert U_s\Vert ^2 ds\right) ,\quad {\mathbb {P}}\text {-a.s.}, \end{aligned}$$

which is possible due to the convergence of $(Y^n,Z^n,U^n)$ in ${{\mathscr {S}}}^p\times L^p(W)\times L^p({\tilde{N}})$. Severini–Egorov’s theorem now permits for a given $\varepsilon >0$ the existence of a set ${\varOmega }_\varepsilon , {\mathbb {P}}({\varOmega }_\varepsilon )>1-\varepsilon$ such that there is a number $N_\varepsilon >0$ with

$$\begin{aligned}\sup _{t\in [0,T]}|Y_t(\omega )-Y^{n_l}_t(\omega )|<1\quad \text {for all }l>N_\varepsilon \text { and }\omega \in {\varOmega }_\varepsilon ,\end{aligned}$$

and the above convergences persist on this set. For given $r>0$ on ${\varOmega }_\varepsilon ^r:={\varOmega }_\varepsilon \cap \left\{ \sup _{t\in [0,T]}|Y_t|\le r\right\}$, we have for $l>N_\varepsilon$,

$$\begin{aligned}&\int _0^T |f(s,Y^{n_l}_s,Z^{n_l}_s,U^{n_l}_s)-f(s,Y_s,Z_s,U_s)|ds\\&\quad \le \int _0^T \left( 2\psi _{r+1}(s)+{\varPhi }(s)\left( |Z^{n_l}_s|+|Z_s|+\Vert U^{n_l}_s\Vert +\Vert U_s\Vert \right) \right) ds<C<\infty , \end{aligned}$$

where *C* may still depend on $\omega \in {\varOmega }_\varepsilon ^r$, and for such $\omega$, the family $\left( s\mapsto (|Z^{n_l}_s\left( \omega )|^2+\Vert U^{n_l}_s(\omega )\Vert ^2\right) _{l\ge 0}\right)$ is uniformly integrable with respect to $\lambda$. Thus, since $f(s,Y^{n_l}_s,Z^{n_l}_s,U^{n_l}_s)\rightarrow f(s,Y_s,Z_s,U_s)$, for $\lambda$-a.a. $s\in [0,T]$ on ${\varOmega }_\varepsilon ^r$, dominated convergence yields

$$\begin{aligned} \lim _{l\rightarrow \infty }\int _t^Tf(s,Y^{n_l}_s,Z^{n_l}_s,U^{n_l}_s)ds= \int _t^Tf(s,Y_s,Z_s,U_s)ds \end{aligned}$$

for $l\rightarrow \infty$ on ${\varOmega }_{\varepsilon }^r$ for all $\varepsilon>0,r>0$. The last identity now even holds on ${\varOmega }^0:=\bigcup _{r>0}\bigcup _{q\ge 1} {\varOmega }_{1/q}^r$ which is an almost sure event. So, the limit of $\int _t^T f^n(s,Y^n_s,Z^n_s,U^n_s)ds$ is uniquely determined as $\int _t^T f(s,Y_s,Z_s,U_s)ds$. Hence, (*Y*, *Z*, *U*) is a solution to BSDE $(\xi ,f)$. $\square$

## Comparison Results

We switch to dimension $d = 1$ and set ${\mathbb {R}}_0 = {\mathbb {R}}{\setminus } \{0\}$ for the following comparison results, generalizing those in [10] to the case of generators that do not have linear growth in the *y*-variable and to an $L^p$-setting for $p>1$. Moreover, in contrast to [10], our proof does not depend on approximation theorems for BSDEs that demand deep measurability results.

### Theorem 2

(Comparison, $p\ge 2$) Let $p, p' \ge 2$ and (*Y*, *Z*, *U*) be the $L^p$-solution to $(\xi ,f)$ and $(Y',Z',U')$ be the $L^{p'}$-solution to $(\xi ',f')$. Furthermore, let *f* and $f'$ satisfy (A 1) and (${\text {A3}}_{\ge 2}$) for the according $p,p'$. If the following assumptions hold

(i): $$\xi \le \xi '$$, $${\mathbb {P}}$$-a.s.,
(ii): $f(s,Y'_s,Z'_s,U'_s)\le f'(s,Y'_s,Z'_s,U'_s)$, for ${\mathbb {P}}\otimes \lambda$-a.a. $(\omega ,s)\in {\varOmega }\times {[0,T]}$ and
(A$$\gamma $$)
for all $u,u'\in L^2(\nu )$ with $u'\ge u$
  29 $$\begin{aligned} f(s,y,z,u)- f(s,y,z,u')\le \int _{{\mathbb {R}}_0}(u'(x)-u(x))\nu (dx), \quad {\mathbb {P}}\otimes \lambda \text {-a.e}, \end{aligned}$$

then for all $t \in [0,T]$, we have ${\mathbb {P}}$-a.s,

$$\begin{aligned} Y_t\le Y'_t. \end{aligned}$$

The same assertion follows from an equivalent formulation for $f'$, requiring $f(s,Y_s,Z_s,U_s) \le f'(s,Y_s,Z_s,U_s)$ and (29) being satisfied for $f'$.

### Proof

The basic idea for this proof was inspired by the one of Theorem 8.3 in [6] and is an extension and simplification of the one in [10].

**Step 1:**

First, note that $(Y,Z,U), (Y',Z',U')$ are solutions in ${{\mathscr {S}}}^2\times L^2(W)\times L^2({\tilde{N}})$.

We use the conditional expectation ${\mathbb {E}}_n$ (see Sect. 2.2) on the BSDEs $(\xi ,f)$ and $(\xi ',f')$ to get (for the BSDE $(\xi ,f)$)

$$\begin{aligned} {\mathbb {E}}_n Y_t&= {\mathbb {E}}_n\xi +\int _t^T {\mathbb {E}}_n f(s,Y_s,Z_s,U_s)ds-\int _t^T {\mathbb {E}}_n Z_s dW_s\\&-\int _{{]t,T]}\times {\mathbb {R}}_0}\chi _{\{1/n\le |x|\}}{\mathbb {E}}_n U_s(x){\tilde{N}}(ds,dx). \end{aligned}$$

Note that here $({\mathbb {E}}_n Z_t)_{t\in {[0,T]}}, ({\mathbb {E}}_n U_t(x))_{t\in {[0,T]}, x\in {\mathbb {R}}_0}$ are considered to be predictable (and $({\mathbb {E}}_n f(t,Y_t,Z_t,U_t))_{t\in {[0,T]}}$ progressively measurable) processes that equal the conditional expectation ${\mathbb {P}}$-a.s. for almost all $t\in {[0,T]}$ (or $\lambda \otimes \nu$ almost every $(t,x)\in {[0,T]}\times {\mathbb {R}}_0$ for the *U*-process). In the case of *Y*, $({\mathbb {E}}_n Y_t)_{t\in {[0,T]}}$ denotes a progressively measurable version of this process. For bounded or nonnegative processes, the construction of such processes can be achieved by using optional projections with parameters (see [18] for optional projections with parameters and [10] for the mentioned construction). In the present case, we are confronted with merely integrable processes: $Y, Z, \int _{{\mathbb {R}}_0}|U_\cdot (x)|^2\nu (dx)$ are integrable and hence also $f(s,Y_s,Z_s,U_s)\in L^1(W)$. The construction of a progressively measurable version for the processes at hand can be found in Lemma 2 and Remark 7 in Appendix.

Moreover, assume for the rest of the proof that the coefficient $\mu$ of *f* is zero: If this was not the case, we could use the transformed variables $({\tilde{Y}}_t,{\tilde{Z}}_t,{\tilde{U}}_t):=e^{\int _0^t\mu (s)ds}(Y_t,Z_t,U_t)$ and $({\tilde{Y}}',{\tilde{Z}}',{\tilde{U}}'):=e^{\int _0^t\mu (s)ds}(Y'_t,Z'_t,U'_t)$.

**Step 2:** We use Tanaka–Meyer’s formula (cf. [22, Chapter 4, Theorem 70 and Corollary 1]) for the squared positive part function $(\cdot )_{+}^2$ to see that for $\eta :=18\beta ^2$,

$$\begin{aligned}&e^{\int _0^t \eta (s)ds}({\mathbb {E}}_nY_t-{\mathbb {E}}_nY'_t)^2_+ \\&\quad =e^{\int _0^T \eta (s)ds}({\mathbb {E}}_n\xi -{\mathbb {E}}_n\xi ')^2_++M(t)\\&\qquad +\int _t^T e^{\int _0^s \eta (\tau )d\tau }\\&\qquad \times \biggl [2({\mathbb {E}}_nY_s-{\mathbb {E}}_nY'_s)_+{\mathbb {E}}_n\left( f(s,Y_s,Z_s,U_s)-f'(s,Y'_s,Z'_s, U'_s)\right) \\&\quad \quad \quad -\chi _{\{{\mathbb {E}}_nY_s-{\mathbb {E}}_nY'_s > 0\}}|{\mathbb {E}}_nZ_s-{\mathbb {E}}_nZ'_s|^2- \eta (s)({\mathbb {E}}_nY_s-{\mathbb {E}}_nY'_s)_+^2\\&\quad \quad \quad -\int _{\{1/n\le |x|\}}\left( ({\mathbb {E}}_nY_s-{\mathbb {E}}_nY'_s+{\mathbb {E}}_nU_s(x)-{\mathbb {E}}_nU'_s(x))^2_+-({\mathbb {E}}_nY_s-{\mathbb {E}}_nY'_s)^2_+\right. \\&\quad \quad \quad \left. -2({\mathbb {E}}_nU_s(x)-{\mathbb {E}}_nU'_s(x))({\mathbb {E}}_nY_s-{\mathbb {E}}_nY'_s)_+\right) \nu (dx)\biggr ]ds. \end{aligned}$$

Here, *M*(*t*) is a stochastic integral term with zero expectation which follows from $Y,Y'\in {{\mathscr {S}}}^2$. Moreover, we used that on the set $\{{\varDelta }^n Y_s > 0\}$ (where ${\varDelta }^n Y:={\mathbb {E}}_n Y-{\mathbb {E}}_n Y'$) we have $(Y_s-Y'_s)_+=|Y_s-Y'_s|$. We denote further differences by ${\varDelta }^n \xi := {\mathbb {E}}_n\xi -{\mathbb {E}}_n\xi ', \,{\varDelta }^n Z := {\mathbb {E}}_nZ-{\mathbb {E}}_nZ'$ and ${\varDelta }^n U:= {\mathbb {E}}_nU-{\mathbb {E}}_nU'$. Observing that the right-hand side increases if we only consider ${\varDelta }^n Y_s > 0$ in the *ds*-integral, we take means to come to

$$\begin{aligned}&{\mathbb {E}}e^{\int _0^t \eta (s)ds}({\varDelta }^n Y_t)^2_+ \\&\quad ={\mathbb {E}}e^{\int _0^T \eta (s)ds}({\varDelta }^n \xi )^2_+ +{\mathbb {E}}\int _t^T e^{\int _0^s \eta (\tau )d\tau }\chi _{\{{\varDelta }^n Y_s > 0\}} \\&\qquad \times \biggl [2({\varDelta }^n Y_s)_+{\mathbb {E}}_n\left( f(s,Y_s,Z_s, U_s)-f'(s,Y'_s,Z'_s, U'_s)\right) -|{\varDelta }^n Z_s|^2- \eta (s)|{\varDelta }^n Y_s|^2\\&\qquad \quad - \int _{\{1/n\le |x|\}}\left( ({\varDelta }^n Y_s+ {\varDelta }^n U_s(x))^2_+-({\varDelta }^n Y_s)^2_+-2({\varDelta }^n U_s(x))({\varDelta }^n Y_s)_+\right) \nu (dx)\biggr ]ds. \end{aligned}$$

We split up the set $\{1/n\le |x|\}$ into

$$\begin{aligned} B_n(s) = B_n(\omega ,s) = \{1/n\le |x|\}\cap \{{\varDelta }^n U_s(x)\ge -{\varDelta }^n Y_s\}\text { and its complement } B_n^c(s). \end{aligned}$$

Taking into account that $\xi \le \xi '\Rightarrow {\mathbb {E}}_n\xi \le {\mathbb {E}}_n \xi '$, we estimate

30 $$\begin{aligned}&{\mathbb {E}}e^{\int _0^t \eta (s)ds}({\varDelta }^n Y_t)^2_+\nonumber \\&\quad \le {\mathbb {E}}\int _t^T e^{\int _0^s \eta (\tau )d\tau }\chi _{\{{\varDelta }^n Y_s> 0\}}\nonumber \\&\qquad \times \biggl [2({\varDelta }^n Y_s)_+{\mathbb {E}}_n\left( f(s,Y_s,Z_s,U_s)-f'(s,Y'_s,Z'_s, U'_s)\right) -|{\varDelta }^n Z_s|^2- \eta (s)|{\varDelta }^n Y_s|^2\nonumber \\&\qquad \quad -\int _{B_n(s)}|{\varDelta }^n U_s(x)|^2\nu (dx)+\int _{B_n^c(s)}\left( ({\varDelta }^n Y_s)^2_++2({\varDelta }^n U_s(x))({\varDelta }^n Y_s)_+\right) \nu (dx)\biggr ]ds. \end{aligned}$$

We focus on $({\varDelta }^n Y_s)_+{\mathbb {E}}_n\left( f(s,Y_s,Z_s,U_s)-f'(s,Y'_s,Z'_s, U'_s)\right)$. By abbreviating ${\varTheta }:= (Y,Z,U)$, ${\varTheta }' := (Y',Z',U')$ and by the assumption $f(s,{\varTheta }') \le f'(s,{\varTheta }')$, we get

31 $$\begin{aligned}&({\varDelta }^n Y_s)_+{\mathbb {E}}_n\left( f(s,{\varTheta }_s)-f'(s,{\varTheta }'_s)\right) \nonumber \\&\quad =({\varDelta }^n Y_s)_+{\mathbb {E}}_n\left( f(s,{\varTheta }_s)-f(s,{\varTheta }'_s)+f(s,{\varTheta }'_s)-f'(s,{\varTheta }'_s)\right) \nonumber \\&\quad \le ({\varDelta }^n Y_s)_+{\mathbb {E}}_n\left( f(s,{\varTheta }_s)-f(s,{\varTheta }'_s)\right) . \end{aligned}$$

Since (${\text {A3}}_{\ge 2}$) implies the Lipschitz property in the *u*- and *z*-variables, we infer, inserting and subtracting the same terms,

32 $$\begin{aligned}&({\varDelta }^n Y_s)_+{\mathbb {E}}_n\left( f(s,Y_s,Z_s,U_s)-f(s,Y'_s,Z'_s, U'_s)\right) \nonumber \\&\quad =({\varDelta }^n Y_s)_+{\mathbb {E}}_n\Big (f(s,Y_s,Z_s,U_s)-f(s,Y'_s,Z'_s, U'_s) \nonumber \\&\qquad +\left( f(s,Y_s,{\mathbb {E}}_n Z_s,{\mathbb {E}}_n U_s)-f(s, Y'_s,{\mathbb {E}}_n Z'_s, {\mathbb {E}}_n U'_s)\right) \nonumber \\&\qquad -\left( f(s,Y_s,{\mathbb {E}}_n Z_s,{\mathbb {E}}_n U_s)-f(s, Y'_s,{\mathbb {E}}_n Z'_s, {\mathbb {E}}_n U'_s)\right) \Big )\nonumber \\&\quad \le ({\varDelta }^n Y_s)_+{\mathbb {E}}_n\left( f(s,Y_s,{\mathbb {E}}_n Z_s,{\mathbb {E}}_n U_s)-f(s,Y_s',{\mathbb {E}}_n Z'_s, {\mathbb {E}}_n U'_s)\right) \nonumber \\&\qquad +({\varDelta }^n Y_s)_+\beta (s)\left( |Z_s-{\mathbb {E}}_n Z_s|+|Z'_s-{\mathbb {E}}_n Z'_s|+\Vert U_s-{\mathbb {E}}_n U_s\Vert +\Vert U'_s-{\mathbb {E}}_n U'_s\Vert \right) . \end{aligned}$$

We estimate, inserting and subtracting terms again, then using (29),

33 $$\begin{aligned}&({\varDelta }^n Y_s)_+{\mathbb {E}}_n\left( f(s,Y_s,{\mathbb {E}}_n Z_s,{\mathbb {E}}_n U_s)-f(s,Y_s',{\mathbb {E}}_n Z'_s, {\mathbb {E}}_n U'_s)\right) \nonumber \\&\quad \le ({\varDelta }^n Y_s)_+{\mathbb {E}}_n\left( f(s,Y_s,{\mathbb {E}}_n Z_s,{\mathbb {E}}_n U_s\chi _{B_n(s)}+{\mathbb {E}}_n U_s\chi _{B^c_n(s)})\right. \nonumber \\&\quad \qquad \left. -f(s,Y_s',{\mathbb {E}}_n Z'_s, {\mathbb {E}}_n U'_s\chi _{B_n(s)}+{\mathbb {E}}_n U_s\chi _{B_n^c(s)})\right) _+\nonumber \\&\qquad +({\varDelta }^n Y_s)_+{\mathbb {E}}_n\left( f(s,Y_s,{\mathbb {E}}_n Z_s,{\mathbb {E}}_n U'_s\chi _{B_n(s)}+{\mathbb {E}}_n U_s\chi _{B^c_n(s)})\right. \nonumber \\&\quad \qquad \left. -f(s,Y_s',{\mathbb {E}}_n Z'_s, {\mathbb {E}}_n U'_s\chi _{B_n(s)}+{\mathbb {E}}_n U'_s\chi _{B_n^c(s)})\right) \nonumber \\&\quad \le ({\varDelta }^n Y_s)_+{\mathbb {E}}_n\left( f(s,Y_s,{\mathbb {E}}_n Z_s,{\mathbb {E}}_n U_s\chi _{B_n(s)}+{\mathbb {E}}_n U_s\chi _{B^c_n(s)})\right. \nonumber \\&\quad \qquad \left. -f(s,Y_s',{\mathbb {E}}_n Z'_s, {\mathbb {E}}_n U'_s\chi _{B_n(s)}+{\mathbb {E}}_n U_s\chi _{B_n^c(s)})\right) _+\nonumber \\&\qquad - \int _{B_n^c(s)}({\varDelta }^n Y_s)_+{\varDelta }^n U_s\nu (dx). \end{aligned}$$

Next we apply Jensen’s inequality in two dimensions for the product of positive random variables and also (${\text {A3}}_{\ge 2}$) and Young’s inequality to arrive at

34 $$\begin{aligned}&({\varDelta }^n Y_s)_+{\mathbb {E}}_n\left( f(s,Y_s,{\mathbb {E}}_n Z_s,{\mathbb {E}}_n U_s\chi _{B_n(s)}+{\mathbb {E}}_n U_s\chi _{B^c_n(s)})\right. \nonumber \\&\qquad \quad \left. -f(s,Y_s',{\mathbb {E}}_n Z'_s, {\mathbb {E}}_n U'_s\chi _{B_n(s)}+{\mathbb {E}}_n U_s\chi _{B_n^c(s)})\right) _+\nonumber \\&\quad \le {\mathbb {E}}_n\left[ {\varDelta }Y_s \left( f(s,Y_s,{\mathbb {E}}_n Z_s,{\mathbb {E}}_n U_s\chi _{B_n(s)}+{\mathbb {E}}_n U_s\chi _{B^c_n(s)})\right. \right. \nonumber \\&\qquad \quad \left. \left. -f(s,Y_s',{\mathbb {E}}_n Z'_s, {\mathbb {E}}_n U'_s\chi _{B_n(s)}+{\mathbb {E}}_n U_s\chi _{B_n^c(s)})\right) \right] _+\nonumber \\&\quad \le {\mathbb {E}}_n\alpha (s)\rho (({\varDelta }Y_s)_+^2)+2{\mathbb {E}}_n\beta (s)^2({\varDelta }Y_s)_+^2+\frac{|{\varDelta }^n Z_s|^2}{4}+\frac{{\mathbb {E}}_n\Vert {\varDelta }^n U_s\chi _{B_n(s)}\Vert ^2}{4}. \end{aligned}$$

Taking together inequalities (31), (32), (33) and (34), we get with Young’s inequality again that

$$\begin{aligned}&({\varDelta }^n Y_s)_+{\mathbb {E}}_n\left( f(s,Y_s,Z_s,U_s)-f'(s,Y'_s,Z'_s, U'_s)\right) \\&\quad \le {\mathbb {E}}_n\alpha (s)\rho (({\varDelta }Y_s)_+^2)+2{\mathbb {E}}_n\beta (s)^2({\varDelta }Y_s)_+^2+\frac{|{\varDelta }^n Z_s|^2}{4}+\frac{{\mathbb {E}}_n\Vert {\varDelta }^n U_s\chi _{B_n(s)}\Vert ^2}{4}\\&\qquad -\int _{B_n^c(s)}({\varDelta }^n Y_s)_+{\varDelta }^n U_s\nu (dx)\\&\qquad +4\beta (s)^2({\varDelta }^n Y_s)^2_+ \\&\qquad +\frac{1}{4}\left( |Z_s-{\mathbb {E}}_n Z_s|^2+|Z'_s-{\mathbb {E}}_n Z'_s|^2+\Vert U_s-{\mathbb {E}}_n U_s\Vert ^2+\Vert U'_s-{\mathbb {E}}_n U'_s\Vert ^2\right) . \end{aligned}$$

Therefore, (30) evolves to

$$\begin{aligned}&{\mathbb {E}}e^{\int _0^t \eta (s)ds}({\varDelta }^n Y_t)^2_+\\&\quad \le \ {\mathbb {E}}\int _t^T e^{\int _0^s \eta (\tau )d\tau }\chi _{\{{\varDelta }^n Y_s> 0\}} \\&\qquad \biggl [2{\mathbb {E}}_n\alpha (s){\rho }(({\varDelta }Y_s)_+^2)+4{\mathbb {E}}_n\beta (s)^2({\varDelta }Y_s)_+^2+\frac{|{\varDelta }^n Z_s|^2}{2}\\&\qquad +\frac{\Vert {\varDelta }^n U_s\chi _{B_n(s)}\Vert ^2}{2} -\int _{B_n^c(s)}2({\varDelta }^n Y_s)_+ {\varDelta }^n U_s(x)\nu (dx)+8\beta (s)^2({\varDelta }^n Y_s)^2_+\\&\qquad +\frac{1}{2}\left( |Z_s-{\mathbb {E}}_n Z_s|^2+|Z'_s-{\mathbb {E}}_n Z'_s|^2+\Vert U_s-{\mathbb {E}}_n U_s\Vert ^2+\Vert U'_s-{\mathbb {E}}_n U'_s\Vert ^2\right) \\&\qquad -|{\varDelta }^n Z_s|^2 -\eta (s)|{\varDelta }^n Y_s|^2-\int _{B_n(s)}|{\varDelta }^n U_s(x)|^2\nu (dx) \\&\qquad +\int _{_{B_n^c(s)}}\left( ({\varDelta }^n Y_s)^2_++2({\varDelta }^n Y_s)_+({\varDelta }^n U_s(x))\right) \nu (dx)\biggr ]ds. \end{aligned}$$

We cancel out terms and end this step with the estimate

35 $$\begin{aligned}&{\mathbb {E}}e^{\int _0^t \eta (s)ds}({\varDelta }^n Y_t)^2_+\nonumber \\&\quad \le \ {\mathbb {E}}\int _t^T e^{\int _0^s \eta (\tau )d\tau } \chi _{\{{\varDelta }^n Y_s> 0\}} \nonumber \\&\qquad \biggl [2{\mathbb {E}}_n\alpha (s){\rho }(({\varDelta }Y_s)_+^2)+4{\mathbb {E}}_n\beta (s)^2({\varDelta }Y_s)_+^2\nonumber \\&\quad \qquad +\frac{1}{2}\left( |Z_s-{\mathbb {E}}_n Z_s|^2+|Z'_s-{\mathbb {E}}_n Z'_s|^2+\Vert U_s-{\mathbb {E}}_n U_s\Vert ^2+\Vert U'_s-{\mathbb {E}}_n U'_s\Vert ^2\right) \nonumber \\&\quad \qquad +8\beta (s)^2({\varDelta }^n Y_s)^2_+-\eta (s)|{\varDelta }^n Y_s|^2+\int _{_{B_n^c(s)}}({\varDelta }^n Y_s)^2_+\nu (dx)\biggr ]ds. \end{aligned}$$

**Step 3:**

We assume without loss of generality that the integrals

$$\begin{aligned} {\mathbb {E}}\int _0^Te^{\int _0^s \eta (\tau )d\tau }\chi _{\{{\varDelta }Y_s> 0\}}\alpha (s){\rho }(({\varDelta }Y_s)_+^2)ds=:\delta _\rho \end{aligned}$$

and

$$\begin{aligned}&{\mathbb {E}}\int _0^Te^{\int _0^s \eta (\tau )d\tau }\chi _{\{{\varDelta }Y_s> 0\}}\beta (s)^2({\varDelta }Y_s)_+^2ds=:\delta _y. \end{aligned}$$

are positive numbers. All other cases would simplify the proof.

Since ${\mathbb {E}}_n Y_s\rightarrow Y_s$ a.s. for all *s*, dominated convergence shows that also

$$\begin{aligned}&{\mathbb {E}}\int _0^Te^{\int _0^s \eta (\tau )d\tau }\left| \chi _{\{{\varDelta }^n Y_s> 0\}}\alpha (s){\rho }(({\varDelta }Y_s)_+^2)-\chi _{\{{\varDelta }^n Y_s> 0\}}\alpha (s){\rho }(({\varDelta }^n Y_s)_+^2)\right| ds, \\&{\mathbb {E}}\int _0^Te^{\int _0^s \eta (\tau )d\tau }\left| \chi _{\{{\varDelta }Y_s> 0\}}\alpha (s){\rho }(({\varDelta }Y_s)_+^2)-\chi _{\{{\varDelta }^n Y_s> 0\}}\alpha (s){\rho }(({\varDelta }^n Y_s)_+^2)\right| ds \end{aligned}$$

and

$$\begin{aligned} {\mathbb {E}}\int _0^Te^{\int _0^s \eta (\tau )d\tau }\left| \chi _{\{{\varDelta }Y_s> 0\}}\alpha (s){\rho }(({\varDelta }Y_s)_+^2)-\chi _{\{{\varDelta }^n Y_s> 0\}}\alpha (s){\rho }(({\varDelta }Y_s)_+^2)\right| ds \end{aligned}$$

converge to zero. For domination, we use

$$\begin{aligned}&{\mathbb {E}}\int _0^Te^{\int _0^s \eta (\tau )d\tau }\alpha (s)\left( {\rho }(({\varDelta }Y_s)_+^2)+{\rho }\Big (\sup _{n\ge 0}{\mathbb {E}}_n({\varDelta }Y_s)_+^2\Big )\right) ds\\&\quad \le e^C\Vert \alpha \Vert _{L^1([0,T])}(1+b\Vert Y\Vert ^2_{{{\mathscr {S}}}^2})\\&\qquad +e^C\Vert \alpha \Vert _{L^1([0,T])}\left( (1+b)(\sup _{n\ge 0}{\mathbb {E}}_n(\sup _{t\in {[0,T]}}{\varDelta }Y_t))^2\right) \\&\quad \le e^C\Vert \alpha \Vert _{L^1([0,T])}(2+5b\Vert Y\Vert ^2_{{{\mathscr {S}}}^2})<\infty , \end{aligned}$$

where we applied that $\int _0^T \eta (s)ds<C$ a.s., Doob’s martingale inequality and that there is $b>0$ such that for $x\ge 0: \rho (x)\le 1+b x$.

For $m\ge 0$ with $\delta _\rho -\frac{1}{m}>0$, let us now choose $N_m\in {\mathbb {N}}$ large enough, such that for $n\ge N_m$:

$$\begin{aligned} {\mathbb {E}}\int _0^Te^{\int _0^s \eta (\tau )d\tau }\left| \chi _{\{{\varDelta }^n Y_s> 0\}}\alpha (s){\rho }(({\varDelta }Y_s)_+^2)-\chi _{\{{\varDelta }^n Y_s> 0\}}\alpha (s){\rho }(({\varDelta }^n Y_s)_+^2)\right| ds<\delta _\rho -\frac{1}{m} \end{aligned}$$

and

$$\begin{aligned} {\mathbb {E}}\int _0^Te^{\int _0^s \eta (\tau )d\tau }\chi _{\{{\varDelta }^n Y_s> 0\}}\alpha (s){\rho }(({\varDelta }^n Y_s)_+^2)ds\ge \delta _\rho -\frac{1}{m}. \end{aligned}$$

For such an *n*, we get that

$$\begin{aligned}&{\mathbb {E}}\int _0^Te^{\int _0^s \eta (\tau )d\tau }\chi _{\{{\varDelta }^n Y_s> 0\}}\alpha (s){\rho }(({\varDelta }Y_s)_+^2)ds\\&\quad \le 2{\mathbb {E}}\int _0^Te^{\int _0^s \eta (\tau )d\tau }\chi _{\{{\varDelta }^n Y_s> 0\}}\alpha (s){\rho }(({\varDelta }^n Y_s)_+^2)ds. \end{aligned}$$

In the same way, one can choose $m,N_m\in {\mathbb {N}}$ also large enough such that for all $n\ge N_m$,

36 $$\begin{aligned}&{\mathbb {E}}\int _0^Te^{\int _0^s \eta (\tau )d\tau }\chi _{\{{\varDelta }^n Y_s> 0\}}\beta (s)^2({\varDelta }Y_s)_+^2ds\nonumber \\&\quad \quad \le 2{\mathbb {E}}\int _0^Te^{\int _0^s \eta (\tau )d\tau }\chi _{\{{\varDelta }^n Y_s> 0\}}\beta (s)^2({\varDelta }^n Y_s)_+^2ds. \end{aligned}$$

Similarly, by martingale convergence ${\mathbb {E}}_n Z_s\rightarrow Z_s$ and a domination argument, we can conclude that for $n\ge N_m$ ($N_m$ may have to be rechosen large enough),

37 $$\begin{aligned}&{\mathbb {E}}\int _0^Te^{\int _0^s \eta (\tau )d\tau }\chi _{\{{\varDelta }^n Y_s> 0\}}| Z_s-{\mathbb {E}}_nZ_s|^2ds \nonumber \\&\quad \le {\mathbb {E}}\int _0^Te^{\int _0^s \eta (\tau )d\tau }\chi _{\{{\varDelta }^n Y_s> 0\}}\beta (s)^2|{\varDelta }^n Y_s|^2ds\quad \text {and}\nonumber \\&{\mathbb {E}}\int _0^Te^{\int _0^s \eta (\tau )d\tau }\chi _{\{{\varDelta }^n Y_s> 0\}}\Vert U_s-{\mathbb {E}}_n U_s\Vert ^2ds \nonumber \\&\quad \le {\mathbb {E}}\int _0^Te^{\int _0^s \eta (\tau )d\tau }\chi _{\{{\varDelta }^n Y_s> 0\}}\beta (s)^2|{\varDelta }^n Y_s|^2ds, \end{aligned}$$

since the left-hand sides tend to zero, while the right-hand sides converge to $\delta _y$. The same estimates hold for $Z'$ and $U'$ as well.

Hence, applying (36) and (37) to (35) yields

38 $$\begin{aligned} {\mathbb {E}}e^{\int _0^t \eta (s)ds}({\varDelta }^n Y_t)^2_+&\le \ {\mathbb {E}}\int _t^T e^{\int _0^s \eta (\tau )d\tau }\chi _{\{{\varDelta }^n Y_s> 0\}}\biggl [4\alpha (s){\rho }(({\varDelta }^n Y_s)_+^2)\nonumber \\&\quad +18\beta (s)^2({\varDelta }^n Y_s)^2_+-\eta (s)|{\varDelta }^n Y_s|^2+\int _{_{B_n^c}}({\varDelta }^n Y_s)^2_+\nu (dx)\biggr ]ds. \end{aligned}$$

**Step 4:**

Bounding $\int _{B_n^c(s)}({\varDelta }^n Y_s)^2_+\nu (dx)$ by $\nu (\{1/n\le |x|\})({\varDelta }^n Y_s)^2_+$ in (38) leads us to

$$\begin{aligned} {\mathbb {E}}e^{\int _0^t \eta (s)ds}({\varDelta }^n Y_t)^2_+&\le \ {\mathbb {E}}\int _t^T e^{\int _0^s \eta (\tau )d\tau }\chi _{\{{\varDelta }^n Y_s> 0\}}\biggl [4\alpha (s){\rho }(({\varDelta }^n Y_s)_+^2)\\&\quad +(\nu (\{1/n\le |x|\})+18\beta (s)^2)({\varDelta }^n Y_s)^2_+-\eta (s)|{\varDelta }^n Y_s|^2\biggr ]ds. \end{aligned}$$

It remains, recalling that $\eta = 18 \beta ^2$,

$$\begin{aligned}&{\mathbb {E}}e^{\int _0^t \eta (s)ds}({\varDelta }^n Y_t)^2_+\\&\quad \le {\mathbb {E}}\int _t^T e^{\int _0^s \eta (\tau )d\tau }\biggl [4\alpha (s){\rho }(({\varDelta }^n Y_s)_+^2)+\nu (\{1/n\le |x|\})({\varDelta }^n Y_s)^2_+\biggr ]ds\\&\quad \le {\mathbb {E}}\int _t^T e^{\int _0^s \eta (\tau )d\tau }\biggl [4(\alpha (s)\vee 1){\left( \rho +\nu (\{1/n\le |x|\})\mathrm {id}\right) }(({\varDelta }^n Y_s)_+^2)ds. \end{aligned}$$

The term $e^{\int _0^T \eta (\tau )d\tau }$ is ${\mathbb {P}}$-a.s. bounded by a constant $C>0$. Thus, by the concavity of $\rho _n:=\rho +\nu (\{1/n\le |x|\})\mathrm {id}$, which satisfies the same assumptions as $\rho$, we arrive at

$$\begin{aligned}&{\mathbb {E}}({\varDelta }^n Y_t)^2_+\le {\mathbb {E}}e^{\int _0^t \eta (s)ds}({\varDelta }^n Y_t)^2_+\le \int _t^T 4C(\alpha (s)\vee 1){\rho _n}({\mathbb {E}}({\varDelta }^n Y_s)_+^2)ds. \end{aligned}$$

Then, the Bihari–LaSalle inequality (Theorem 5) shows that ${\mathbb {E}}({\varDelta }^n Y_t)^2_+=0$ for all $t\in {[0,T]}$.

**Step 5:**

Steps 1-4 granted that ${\mathbb {E}}_n Y_t \le {\mathbb {E}}_n Y_t'$ for *n* greater than a certain value, for all $t \in [0,T]$. By martingale convergence, ${\mathbb {E}}_n Y_t$ converges almost surely to the solution $Y_t$ of $(\xi ,f)$ at time *t* and ${\mathbb {E}}_n Y_t'$ converges to the solution $Y_t'$ of $(\xi ',f')$. Hence, in the limit we have $Y_t \le Y_t',$ and the theorem is proved. $\square$

In Theorem 3, we state a version of the above theorem for the case $1<p<2$. The difference to Theorem 2 is that here we cannot compare the generators on the solution only. If one wants to keep the comparison of the generators on the solution, but accepts a slightly stronger condition than (29), given as ($\mathrm {H}_{\mathrm {comp}}$)in [13],

(A$$\gamma $$’)
   $$f(s,y,z,u)- f(s,y,z,u')\le \int _{{\mathbb {R}}_0} (u(x) - u'(x)) \gamma _t(x) \nu (dx), \quad {\mathbb {P}}\otimes \lambda $$-a.e.
  for a predictable process $\gamma = \gamma ^{y,z,u,u'}$, such that $-1 \le \gamma _t(x)$ and $|\gamma _t(u)| \le \vartheta (u)$, where $\vartheta \in L^2(\nu )$,

then the proof of [13, Proposition 4] can also be conducted for generators satisfying the conditions (${\text {A3}}_{\ge 2}$) or (${\text {A3}}_{< 2}$).

### Theorem 3

(Comparison, $p>1$) Let $p, p' > 1$ and (*Y*, *Z*, *U*) be the $L^p$-solution to $(\xi ,f)$ and $(Y',Z',U')$ be the $L^{p'}$-solution to $(\xi ',f')$. Furthermore, let *f* and $f'$ satisfy (A 1) and (${\text {A3}}_{\ge 2}$) or (${\text {A3}}_{< 2}$) for the according $p,p'$. If the following assumptions hold

(i): $$\xi \le \xi '$$, $${\mathbb {P}}$$-a.s.,
(ii): $f(s,y,z,u)\le f'(s,y,z,u)$, for all $(y,z,u)\in {\mathbb {R}}\times {\mathbb {R}}\times L^2(\nu ),$ for ${\mathbb {P}}\otimes \lambda$-a.a. $(\omega ,s)\in {\varOmega }\times {[0,T]}$ and
(A$$\gamma $$)
for all $u,u'\in L^2(\nu )$ with $u'\ge u$
  39 $$\begin{aligned} f(s,y,z,u)- f(s,y,z,u')\le \int _{{\mathbb {R}}_0}(u'(x)-u(x))\nu (dx), \quad {\mathbb {P}}\otimes \lambda \text {-a.e}, \end{aligned}$$

then for all $t \in [0,T]$, we have ${\mathbb {P}}$-a.s.,

$$\begin{aligned} Y_t\le Y'_t. \end{aligned}$$

The same assertion follows from an equivalent formulation for $f'$, requiring that (39) holds for $f'$.

### Proof

We approximate the generators and terminal conditions by $\xi _n:=\frac{n}{|\xi |\vee n}\xi , \xi '_n:=\frac{n}{|\xi '|\vee n}\xi '$ and

$$\begin{aligned}&f_n(t,y,z,u):=\frac{n}{|f(t,y,z,u)|\vee n}f(t,y,z,u),\\&f'_n(t,y,z,u):=\frac{n}{|f'(t,y,z,u)|\vee n}f'(t,y,z,u). \end{aligned}$$

This procedure preserves order relations. Furthermore, the generators $f_n, f'_n$ satisfy (A 1), (${\text {A3}}_{\ge 2}$) or (${\text {A3}}_{< 2}$) with respect to their coefficients. Also (39) remains satisfied for $f_n$.

Thus, the solutions $Y_n$ and $Y'_n$ of all these equations satisfy for all $t\in {[0,T]}$ $Y_{n,t}\le Y'_{n,t}, {\mathbb {P}}\text {-a.s.}$ since (by the boundedness of $\xi _n,\xi '_n,f_n, f'_n$ and since Remark 2, (a) implies (${\text {A3}}_{\ge 2}$) for $p=2$ for the $f_n, f'_n$) they are also $L^2$-solutions. In the following steps, we will show convergence of those solutions to *Y* in ${{\mathscr {S}}}^{p\wedge 2}$ and $Y'$ in ${{\mathscr {S}}}^{p'\wedge 2}$. Since for $p>2$ (or $p'>2$) the according solution is also an $L^2$-solution, convergence in ${{\mathscr {S}}}^2$ will suffice. Therefore, we will concentrate in the sequel on the case $p\le 2$ for *Y* (the case for $p'$ and $Y'$ works the same way).

**Step 1:**

Consider the difference of the BSDEs for $Y_n$ and *Y* (the convergence $Y'_n\rightarrow Y'$ can be shown in exactly the same way), with solutions $(Y_n,Z_n,U_n)$ and (*Y*, *Z*, *U*),

$$\begin{aligned} Y_{n,t}-Y_t=&\xi _n-\xi +\int _t^T\left( f_n(s,Y_{n,s},Z_{n,s},U_{n,s}) -f(s,Y_s,Z_s,U_s)\right) ds \\&-\int _t^T(Z_{n,s}-Z_s)dW_s\\&-\int _{]t,T]\times {\mathbb {R}}_0}\left( U_{n,s}(x)-U_s(x)\right) {\tilde{N}}(ds,dx), \end{aligned}$$

which can be written as a BSDE for $(Y_n-Y, Z_n-Z, U_n-U)$ using the generator

$$\begin{aligned} (s,y,z,u)\mapsto f_n(s,y+Y_s,z+Z_s,u+U_s)-f(s,Y_s,Z_s,U_s). \end{aligned}$$

Then, Proposition 4, whose assumptions (A 1), (${\text {a3}}_{< 2}$) are met for $\xi _n-\xi$ and this generator, yields

$$\begin{aligned}&\Vert Y_n-Y\Vert _{{{\mathscr {S}}}^p}^p+\Vert Z_n-Z\Vert _{L^p(W)}^p+\Vert U_n-U\Vert _{L^p({\tilde{N}})}\\&\quad \le h\left( {\mathbb {E}}|\xi _n-\xi |^p+{\mathbb {E}}\left[ \int _0^T\left| f_n (s,Y_{s},Z_{s},U_{s})-f(s,Y_s,Z_s, U_s)\right| ds\right] ^p\right) . \end{aligned}$$

Clearly $\xi _n$ converges to $\xi$ in $L^p$. To obtain the desired convergence of $Y_n$ to *Y*, we need that ${\mathbb {E}}\left[ \int _0^T\left| f_n (s,Y_{s},Z_{s},U_{s})-f(s,Y_s,Z_s, U_s)\right| ds\right] ^p\rightarrow 0$.

This expression can be written as

$$\begin{aligned}&{\mathbb {E}}\left[ \int _0^T\left| f_n (s,Y_{s},Z_{s},U_{s})-f(s,Y_s,Z_s, U_s)\right| ds\right] ^p\\&\quad ={\mathbb {E}}\left[ \int _0^T\left| f_n (s,Y_{s},Z_{s},U_{s})-f(s,Y_s ,Z_s, U_s)\right| \chi _{\{|f(s,Y_s,Z_s, U_s)|>n\}}ds\right] ^p, \end{aligned}$$

which we can bound by

$$\begin{aligned} {\mathbb {E}}\left[ \int _0^T2\left| f(s,Y_s,Z_s, U_s)\right| \chi _{\{|f(s,Y_s ,Z_s, U_s)|>n\}}ds\right] ^p. \end{aligned}$$

To show that the above sequence tends to 0, it remains to show that

$$\begin{aligned} {\mathbb {E}}\left[ \int _0^T\left| f(s,Y_s,Z_s, U_s)\right| ds\right] ^p<\infty , \end{aligned}$$

which we will do by a method appearing in the Brownian setting for scalar BSDEs in [3, Remark 4.3]. This will be the content of the next step.

**Step 2:**

The limit version of Itô’s formula (see [13, Corollary 1] and the estimate [13, Lemma 9]), applied to the modulus function $|\cdot |$ and the BSDE $(\xi ,f)$, together with immediate estimates yields

$$\begin{aligned} |Y_0|\le&|\xi |+\int _0^T\mathrm {sign}(Y_s)\left( f(s,Y_s,Z_s)-f_0(s)\right) ds+\int _0^T\mathrm {sign}(Y_s)f_0(s)ds\\&-p\int _0^T\mathrm {sign}(Y_s)Z_sdW_s-p\int _{]0,T]\times {\mathbb {R}}_0}\mathrm {sign}(Y_{s-})U_s(x){\tilde{N}}(ds,dx). \end{aligned}$$

Hence, for the positive and negative part of $\mathrm {sign}(Y_s)\left( f(s,Y_s,Z_s)-f_0(s)\right)$ we get

$$\begin{aligned}&\int _0^T\left[ \mathrm {sign}(Y_s)\left( f(s,Y_s,Z_s)-f_0(s)\right) \right] _-ds\\&\quad \le |\xi |+\int _0^T\left[ \mathrm {sign}(Y_s)\left( f(s,Y_s,Z_s)-f_0(s)\right) \right] _+ds+\int _0^T\left| f_0(s)\right| ds\\&\qquad -p\int _0^T\mathrm {sign}(Y_s)Z_sdW_s-p\int _{]0,T]\times {\mathbb {R}}_0}\mathrm {sign}(Y_{s-})U_s(x){\tilde{N}}(ds,dx). \end{aligned}$$

Adding the integral $\int _0^T\left[ \mathrm {sign}(Y_s)\left( f(s,Y_s,Z_s)-f_0(s)\right) \right] _+ds$ on both sides, we get

$$\begin{aligned}&\int _0^T\left| f(s,Y_s,Z_s)-f_0(s)\right| ds\\&\quad \le |\xi |+2\int _0^T\left[ \mathrm {sign}(Y_s)\left( f(s,Y_s,Z_s)-f_0(s)\right) \right] _+ds+\int _0^T\left| f_0(s)\right| ds\\&\qquad -p\int _0^T\mathrm {sign}(Y_s)Z_sdW_s-p\int _{]0,T]\times {\mathbb {R}}_0}\mathrm {sign}(Y_{s-})U_s(x){\tilde{N}}(ds,dx). \end{aligned}$$

By (${\text {A3}}_{< 2}$), we come to

$$\begin{aligned}&\int _0^T\left| f(s,Y_s,Z_s)-f_0(s)\right| ds\\&\quad \le |\xi |+2\int _0^T \chi _{\{ Y_s \ne 0\}} \\&\qquad \left( \alpha (s)\rho \left( |Y_s|^p\right) |Y_s|^{1-p}+\mu (s)|Y_s|+\beta _1(s)|Z _s|+\beta _2(s)\Vert U_s\Vert \right) ds \\&\qquad +\int _0^T\left| f_0(s)\right| ds\\&\qquad -p\int _0^T\mathrm {sign}(Y_s)Z_sdW_s-p\int _{]0,T]\times {\mathbb {R}}_0}\mathrm {sign}(Y_{s-})U_s(x){\tilde{N}}(ds,dx). \end{aligned}$$

Since $\lim _{x\rightarrow 0}\tfrac{\rho (x^p)}{x^{p-1}}=0$, there is a value $r>0$ such that $\tfrac{\rho (x^p)}{x^{p-1}}\le 1$, for $x \le r$. Additionally, from the concavity of $\rho$ it follows that for $x\ge r$ we have $\rho (x)\le \tfrac{\rho (r)}{r}x$. This means that for $x\ge 0$,

$$\begin{aligned} \rho (x^p)x^{1-p}\le 1\chi _{\{x\le r\}}+\frac{\rho (r)}{r}x\chi _{\{x\ge r\}}\le 1+\frac{\rho (r)}{r}x. \end{aligned}$$

With this inequality for the function $\rho$, we arrive at

$$\begin{aligned}&\int _0^T\left| f(s,Y_s,Z_s)-f_0(s)\right| ds\\&\quad \le |\xi |+2\int _0^T\alpha (s)ds \\&\qquad +2\int _0^T\left( \left( \alpha (s)\frac{\rho (r)}{r}+\mu (s)\right) |Y_s|+\beta _1(s)|Z _s|+\beta _2(s)\Vert U_s\Vert \right) ds+\int _0^T\left| f_0(s)\right| ds\\&\qquad +p\left| \int _0^T\mathrm {sign}(Y_s)Z_sdW_s\right| +p\left| \int _{]0,T]\times {\mathbb {R}}_0}\mathrm {sign}(Y_{s-})U_s(x){\tilde{N}}(ds,dx)\right| . \end{aligned}$$

We take the *p*th power and expectations and then estimate with a constant $c>0$,

40 $$\begin{aligned}&{\mathbb {E}}\left[ \int _0^T\left| f(s,Y_s,Z_s)-f_0(s)\right| ds\right] ^p\nonumber \\&\quad \le c{\mathbb {E}}\Biggl (1+|\xi |^p+\left[ \int _0^T\left( \alpha (s)\frac{\rho (r)}{r}+\mu (s)\right) |Y_s|ds\right] ^p \nonumber \\&\qquad \quad +\left[ \int _0^T\beta _1(s)|Z _s|ds\right] ^p+\left[ \int _0^T\beta _2(s)\Vert U_s\Vert ds\right] ^p\nonumber \\&\quad \qquad +\left[ \int _0^T\left| f_0(s)\right| ds\right] ^p +\left| \int _0^T\mathrm {sign}(Y_s)Z_sdW_s\right| ^p \nonumber \\&\qquad \quad +\left| \int _{]0,T] \times {\mathbb {R}}_0}\mathrm {sign}(Y_{s-})U_s(x){\tilde{N}}(ds,dx)\right| ^p\Biggr ). \end{aligned}$$

By the Cauchy–Schwarz inequality,

$$\begin{aligned}&{\mathbb {E}}\left[ \int _0^T\beta _1(s)|Z _s|ds\right] ^p+{\mathbb {E}}\left[ \int _0^T\beta _2(s)\Vert U_s\Vert ds\right] ^p\\&\quad \le {\mathbb {E}}\left[ \int _0^T \beta _1(s)^2ds\right] ^\frac{p}{2}\left[ \int _0^T |Z_s|^2ds\right] ^\frac{p}{2}+{\mathbb {E}}\left[ \int _0^T \beta _2(s)^2ds\right] ^\frac{p}{2}\left[ \int _0^T \Vert U_s\Vert ^2ds\right] ^\frac{p}{2}\\&\quad \le c_1{\mathbb {E}}\left( \left[ \int _0^T |Z_s|^2ds\right] ^\frac{p}{2}+\left[ \int _0^T \Vert U_s\Vert ^2ds\right] ^\frac{p}{2}\right) <\infty , \end{aligned}$$

for some constant $c_1>0$, and further,

$$\begin{aligned}&{\mathbb {E}}\left[ \int _0^T\left( \alpha (s)\frac{\rho (r)}{r}+\mu (s)\right) |Y_s|ds\right] ^p \\&\quad \le {\mathbb {E}}\sup _{s\in {[0,T]}}|Y_s|^p\left[ \int _0^T\left( \alpha (s)\frac{\rho (r)}{r}+\mu (s)\right) ds\right] ^p\\&\quad \le c_2{\mathbb {E}}\sup _{s\in {[0,T]}}|Y_s|^p<\infty , \end{aligned}$$

for some $c_2>0$. The finiteness of ${\mathbb {E}}\left| \int _0^T\mathrm {sign}(Y_s)Z_sdW_s\right| ^p$ follows from the Burkholder–Davis–Gundy inequality and the one of ${\mathbb {E}}\left| \int _{]0,T]\times {\mathbb {R}}_0}\mathrm {sign}(Y_{s-})U_s(x){\tilde{N}}(ds,dx)\right| ^p$ follows from [17, Theorem 3.2]. As all terms of the right-hand side in (40) are now finite, we get that

$$\begin{aligned} {\mathbb {E}}\left[ \int _0^T\left| f(s,Y_s,Z_s,U_s)-f_0(s)\right| ds\right] ^p<\infty . \end{aligned}$$

The finiteness of ${\mathbb {E}}\left[ \int _0^T|f_0(s)|ds\right] ^p$ implies that ${\mathbb {E}}\left[ \int _0^T|f(s,Y_s,Z_s,U_s)|ds\right] ^p<\infty$, as desired. Therefore, with step 1 we conclude that the convergence

$$\begin{aligned} {\mathbb {E}}\left[ \int _0^T|f_n(s,Y_s,Z_s,U_s)-f(s,Y_s,Z_s,U_s)|ds\right] ^p\rightarrow 0 \end{aligned}$$

takes place which proves that $Y_n\rightarrow Y$ in ${{\mathscr {S}}}^{p\wedge 2}$.

Then, for all *t*,

$$\begin{aligned} Y_{n,t}\rightarrow Y_{t},\quad Y'_{n,t}\rightarrow Y'_{t},\quad {\mathbb {P}}\text {-a.s.,} \end{aligned}$$

and $$Y_t\le Y'_t$$, $${\mathbb {P}}$$-a.s. follows. $$\square $$

## Appendix

### 6.1 Inequalities

#### Theorem 4

(Young’s inequality) For $R > 0$ and $a,b \in {\mathbb {R}}$, we can bound the product

$$\begin{aligned} ab = aR^{-\frac{1}{p}} R^{\frac{1}{p}} b \le \frac{a^p}{pR}+\frac{b^qR^{\frac{q}{p}}}{q}\, \end{aligned}$$

with $$p,q>1$$ satisfying $$\frac{1}{p}+\frac{1}{q}=1$$.

**The Bihari–LaSalle inequality** For the Bihari–LaSalle inequality, we refer to [16, pp. 45–46]. Here, we formulate a backward version of it which has been applied in [30]. The proof is analogous to that in [16].

#### Theorem 5

Let $c>0.$ Assume that $\rho : [0,\infty [ \rightarrow [0,\infty [$ is a continuous and nondecreasing function such that $\rho (x) >0$ for all $x>0.$ Let *K* be a nonnegative, integrable Borel function on [0, *T*] and *y* a nonnegative, bounded Borel function on [0, *T*],  such that

$$\begin{aligned} y(t)&\le c + \int _t^T K(s) \rho (y(s)) ds. \end{aligned}$$

Then it holds that

$$\begin{aligned} y(t) \le G^{-1} \left( G(c) + \int _t^T K(s) ds \right) \end{aligned}$$

for all $t \in [0,T]$ such that $G(c) + \int _t^T K(s) ds \in \mathrm{dom}(G^{-1}).$ Here,

$$\begin{aligned} G(x) := \int _1^x \frac{dr}{\rho (r)}, \end{aligned}$$

and $G^{-1}$ is the inverse function of *G*.

Furthermore, if for an $\epsilon > 0$, $\int _0^\epsilon \frac{dr}{\rho (r)} = \infty$, then

$$\begin{aligned}y(t)=0\end{aligned}$$

for all $$t\in [0,T]$$.

#### Remark 6

As a special case, we have Gronwall’s inequality: If $\rho (r)=r$ for $r\in [0,\infty [,$ we get

$$\begin{aligned} y(t)\le c e^{\int _t^T K(s) ds}. \end{aligned}$$

### Construction of Predictable and Progressively Measurable Versions

We will use the next lemma for the construction of progressively measurable versions of conditional expectations necessary for the proof of Theorem 2. To prepare it, we need the following definitions, appearing also in [10, Definition 3.2] :

Assume that $\mathbb {F}:= \left( {{\mathscr {F}}}^{[s]}\right) _{s\in [0,\infty [}$ is built using the definitions from Sect. 2.2, where $[\cdot ]$ denotes the floor function. Recall that ${{\mathscr {P}}}$ denotes the predictable $\sigma$-algebra according to the filtration $({{\mathscr {F}}}_t)_{t\in [0,T]}$. Let $(V,{{\mathscr {V}}},\mu )$ be a $\sigma$-finite measure space and $K:{\varOmega }\times {[0,T]}\times V\rightarrow {\mathbb {R}}$ be a bounded, ${{\mathscr {P}}}\otimes {{\mathscr {V}}}$-measurable process. Define  to be the optional projection of the process

$$\begin{aligned} {[0,\infty [}\times {\varOmega }\times {[0,T]}\times V \rightarrow&\ \ {\mathbb {R}},\\ (s,\omega ,t,x)\mapsto&\ K(\omega ,t,x) \end{aligned}$$

in the variables $(s,\omega )$ with respect to $\mathbb {F},$ and with parameters (*t*, *x*). For each $n\ge 0$, assume that the filtration $\mathbb {F}^n :=\left( {{\mathscr {F}}}_t^n\right) _{t\in {[0,T]}}$ is given by ${{\mathscr {F}}}_t^n:={{\mathscr {F}}}_t\cap {{\mathscr {F}}}^n.$ Let $K_n$ be the predictable projection of

$$\begin{aligned} (\omega ,t,x)\mapsto ^{o,\mathbb {F}} K(n,\omega ,t,x) \end{aligned}$$

with respect to $\mathbb {F}^n$ with parameter *x*.

The reason for using the family of filtrations $\left( {{\mathscr {F}}}^{[s]}\right) _{s\in [0,\infty [}$ instead of the sequence $({{\mathscr {F}}}^n)_{n=0}^\infty$ from Sect. 2.2 is that one can apply known measurability results w.r.t. right continuous filtrations instead of proving measurability here directly. Indeed, the optional projection  defined above is jointly measurable in $(s,\omega ,t,x).$ For this, we refer to [18], where predictable and optional projections of random processes depending on parameters were considered, and their uniqueness up to indistinguishability was shown.

It follows that for all $(t,x) \in [0,T] \times V$,

Then, since *K* is $\left( {{\mathscr {F}}}_t\right) _{t\in {[0,T]}}$-predictable, for all $n\ge 0$, $t\in {[0,T]}$ and all $x \in V$, it holds that

41 $$\begin{aligned} K_n(t,x)={\mathbb {E}}_n K(t,x), \quad {\mathbb {P}}\text {-a.s.} \end{aligned}$$

Hence, $K_n(t,x)$ is a jointly measurable version of ${\mathbb {E}}_n K(t,x)$ which is $\left( {{\mathscr {F}}}_t^n\right) _{t\in {[0,T]}}$-predictable, so in particular it is predictable.

To extend the above construction to unbounded processes *K*, we have the following result:

#### Lemma 2

Let $(V,{{\mathscr {V}}},\mu )$ be a $\sigma$-finite measure space and $K:{\varOmega }\times {[0,T]}\times V\rightarrow {\mathbb {R}}$ be a ${{\mathscr {P}}}\otimes {{\mathscr {V}}}$-measurable process such that for $\lambda \otimes \mu$-almost all $(t,x)\in {[0,T]}\times V$,

$$\begin{aligned} {\mathbb {E}}|K(t,x)|<\infty . \end{aligned}$$

There is a ${{\mathscr {P}}}\otimes {{\mathscr {V}}}$-measurable process $\left( K_n(t,x)\right) _{(t,x)\in [0,T]\times V}$, and

$$\begin{aligned}K_n(\omega ,t,x)={\mathbb {E}}_n K(\omega ,t,x), {\mathbb {P}}\text {-a.s. for }\lambda \otimes \mu \text {-a.a. }(t,x)\in [0,T]\times V.\end{aligned}$$

#### Proof

For $m \ge 1$, let $K^m:=\frac{m}{|K|\vee m}K$ be a bounded approximation for *K*. For each *m*, we can construct the process $K^m_n$, as described above the lemma. We intend to let $m\rightarrow \infty$. By the definition of the optional and predictable projections, we have that for all $(t,x) \in [0,T] \times V$,

$$\begin{aligned} K^m_n(t,x)={\mathbb {E}}_{t-}{\mathbb {E}}_n K^m(t,x),\quad {\mathbb {P}}\text {-a.s.,} \end{aligned}$$

where we emphasize that ${\mathbb {E}}_{t-}$ is the conditional expectation with respect to the $\sigma$-algebra ${{\mathscr {F}}}_{t-}=\sigma \left( \bigcup _{0\le s<t}{{\mathscr {F}}}_s\right)$ and ${\mathbb {E}}_n$ denotes the expectation with respect to the $\sigma$-algebra ${{\mathscr {F}}}^n$. Using the assumption ${\mathbb {E}}|K(t,x)|<\infty$ for $\lambda \otimes \mu$-a.a. (*t*, *x*), we find by dominated convergence that for a set $A\in {{\mathscr {B}}}([0,T])\otimes {{\mathscr {V}}}$ with $(\lambda \otimes \mu )(A)<\infty$,

$$\begin{aligned}&\int _A {\mathbb {E}}\left( |K^{m_1}_n(t,x)-K^{m_2}_n(t,x)|\wedge 1\right) (\lambda \otimes \mu )(dt, dx)\\&\quad =\int _A {\mathbb {E}}\left( |{\mathbb {E}}_{t-}{\mathbb {E}}_n K^{m_1}(t,x)-{\mathbb {E}}_{t-}{\mathbb {E}}_nK^{m_2}(t,x)|\wedge 1\right) (\lambda \otimes \mu )(dt, dx)\\&\quad \le \int _A {\mathbb {E}}\left( |K^{m_1}(t,x)-K^{m_2}(t,x)|\wedge 1\right) (\lambda \otimes \mu )(dt, dx) \rightarrow 0\quad \text {as }m_2>m_1\rightarrow \infty . \end{aligned}$$

Since $(V,{{\mathscr {V}}},\mu )$ is $\sigma$-finite, it follows that the sequence $(K^m_n)_{m\ge 1}$ converges also locally in the measure ${\mathbb {P}}\otimes \lambda \otimes \mu$, from which we then infer that we can extract a subsequence $(K^{m_k}_n)_{k\ge 1}$ that converges ${\mathbb {P}}\otimes \lambda \otimes \mu$-a.e. Without loss of generality, we assume that $(K^m_n)_{m\ge 1}$ itself converges ${\mathbb {P}}\otimes \lambda \otimes \mu$-a.e. This means that one can find an ${{\mathscr {F}}}\otimes {{\mathscr {B}}}([0,T])\otimes {{\mathscr {V}}}$-measurable process ${{\tilde{K}}}_n$ and a set $M\in {{\mathscr {F}}}\otimes {{\mathscr {B}}}([0,T])\otimes {{\mathscr {V}}}$ with ${\mathbb {P}}\otimes \lambda \otimes \mu \left( ({\varOmega }\times {[0,T]}\times V){\setminus } M\right) =0$ such that for all $(\omega ,t,x)\in M$, $\lim _{m\rightarrow \infty }K^m_n(\omega ,t,x)={{\tilde{K}}}_n(\omega ,t,x)$. On the $\sigma$-algebra

$$\begin{aligned} ({{\mathscr {P}}}\otimes {{\mathscr {V}}})_M:=\left\{ A\cap M : A\in {{\mathscr {P}}}\otimes {{\mathscr {V}}}\right\} , \end{aligned}$$

we have that the process ${{\tilde{K}}}_n$ restricted to the set *M* is the limit of the processes $(K^m_n)$ restricted to *M* and is therefore $({{\mathscr {P}}}\otimes {{\mathscr {V}}})_M$-measurable. In the sense of [25, Theorem 1], we can then extend the restriction of the process ${{\tilde{K}}}_n$ to a ${{\mathscr {P}}}\otimes {{\mathscr {V}}}$-measurable process $K_n$.

To show that the process $K_n$ satisfies condition (41) for $\lambda \otimes \mu$-a.a. $(t,x)\in [0,T]\times V$, one can use the dominated convergence theorem for conditional expectations,

$$\begin{aligned} K_n(t,x)&=\lim _{m\rightarrow \infty } K^m_n(t,x)=\lim _{m\rightarrow \infty }{\mathbb {E}}_{t-}{\mathbb {E}}_n K^m(t,x) \\&={\mathbb {E}}_{t-}{\mathbb {E}}_n\lim _{m\rightarrow \infty } K^m(t,x)={\mathbb {E}}_{t-}{\mathbb {E}}_n K(t,x), \end{aligned}$$

where the latter equals ${\mathbb {E}}_nK(t,x), \lambda \otimes \mu$-a.e. since *K* is a predictable process. $\square$

#### Remark 7

(i) A similar proof as above can be done if one considers optional processes instead of predictable ones. If the process *K* does not depend on an additional parameter set *V* and is such that ${\mathbb {E}}|K(t)|<\infty$, for all *t*, then again very similar arguments as in the above lemma can be used to find that there is a progressively measurable process $K_n$ with ${\mathbb {E}}_nK(t)=K_n(t)$ for all $t\in [0,T]$. (This case is the one needed for a progressively measurable version of $({\mathbb {E}}_n Y_t)_{t\in [0,T]}$ in the proof of Theorem 2.)
(ii) A different way of proving Lemma 2 is to represent the process *K* by a functional $F^K:D[0,T]\times [0,T]\times V$, where *D*[0, *T*] denotes the space of càdlàg functions on [0, *T*] endowed with the $\sigma$-algebra generated by the projection maps $p_t:D[0,T]\rightarrow {\mathbb {R}}, \mathrm {x}\mapsto \mathrm {x}_t$. The measure we consider on this sigma field is the pushforward measure ${\mathbb {P}}_X$ of the Lévy process *X*, given by the trajectory mapping $X:{\varOmega }\rightarrow D[0,T], \omega \mapsto X(\omega )$. We have that $K(t,x)=F^K(X,t,x)$ up to indistinguishability. (For details on this representation, see [27].) Denoting the Lévy process with cutoff Lévy measure again by $X^n$ (see Sect. 2.2), one can then show that the process *G* given by
  $$\begin{aligned} G(t,x)={\left\{ \begin{array}{ll} {\mathbb {E}}F^K(h+X-X^n,t,x)\Big |_{h=X^n}, &{}\text {whenever the expectation exists and is finite,}\\ 0, &{}\text {else},\end{array}\right. } \end{aligned}$$
  possesses a predictable version that satisfies the assertion of Lemma 2.
